# Identifying urban low-altitude traffic corridors: A computational fluid dynamics simulation approach

**DOI:** 10.1371/journal.pone.0357628

**Published:** 2026-09-28

**Authors:** Zhipeng Liu, Zhizhou Wu, Jizhao Wang, Bin Pan, Wenxin Jiang

**Affiliations:** 1 School of Traffic and Transportation Engineering, Xinjiang University, Urumqi, China; 2 College of Transportation, Tongji University, Shanghai, China; 3 School of Mechanical Engineering, Xinjiang University, Urumqi, China; Southwest Jiaotong University, CHINA

## Abstract

With the rapid expansion of unmanned aerial vehicle (UAV) applications in urban logistics, inspection, and emergency services, structured low-altitude networks are increasingly needed to support strategic airspace management. This paper proposes a computational fluid dynamics (CFD)-inspired method for identifying urban low-altitude traffic corridors (ULATCs). Rather than predicting atmospheric wind or actual UAV traffic dynamics, the method uses PhiFlow to construct synthetic incompressible velocity fields constrained by buildings and restricted areas. Multidirectional streamline tracing and geometric post-processing are then used to extract smooth, obstacle-avoiding candidate corridor centerlines. In a 5×5 km^2^ case study in Shenzhen, China, the method identified 10 principal corridors with a mean individual length of 4,904 m and a unique network length of 40.271 km. Compared with the Voronoi–A* and Risk-GNG baseline methods, CFD-FHVCT achieved a 500-m coverage ratio of 93.55% with the shortest unique network length and the highest length-weighted straightness. Path-planning results for six origin–destination pairs further showed that the corridor-constrained paths had 42.3% lower total curvature, despite a 14.6% increase in path length, while mean flight time decreased by 2.2%. These results indicate that the identified network can support smoother trajectories with lower maneuvering demand. Overall, CFD-FHVCT provides a feasible computational framework for strategic urban low-altitude corridor identification and network design, with the synthetic velocity field used to guide corridor extraction rather than to predict physical wind or actual UAV traffic dynamics.

## 1. Introduction

With the rapid advancement of unmanned aerial vehicle (UAV) technology, its applications have expanded substantially across commercial delivery, logistics transportation, and public service sectors [1–3]. For example, by March 2024, China’s food delivery platform Meituan had completed more than 250,000 urban drone delivery orders, reducing average delivery times by over 50%. As demand for low-altitude missions and operations continues to increase, large-scale UAV operations place growing demands on safe operation, reliable communication, and efficient management within limited urban airspace [4–6].

The management of low-altitude airspace remains at an exploratory stage, with existing studies primarily focusing on four categories of airspace structures: free-flight [7,8], hierarchical structures based on heading and altitude [8,9], urban geography-based zoning [10,11], and corridor-based structures [12], as illustrated in Fig 1. Among these approaches, corridor-based airspace design establishes a structured urban low-altitude airspace network that provides UAVs with predefined flight routes. This design reduces potential conflicts and management complexity while enabling the concentrated deployment of communication and sensing infrastructure along critical flight paths. By avoiding extensive infrastructure deployment and the associated increase in costs, corridor-based airspace management is widely regarded as a promising solution for low-altitude airspace control [13,14].

**Fig 1:**
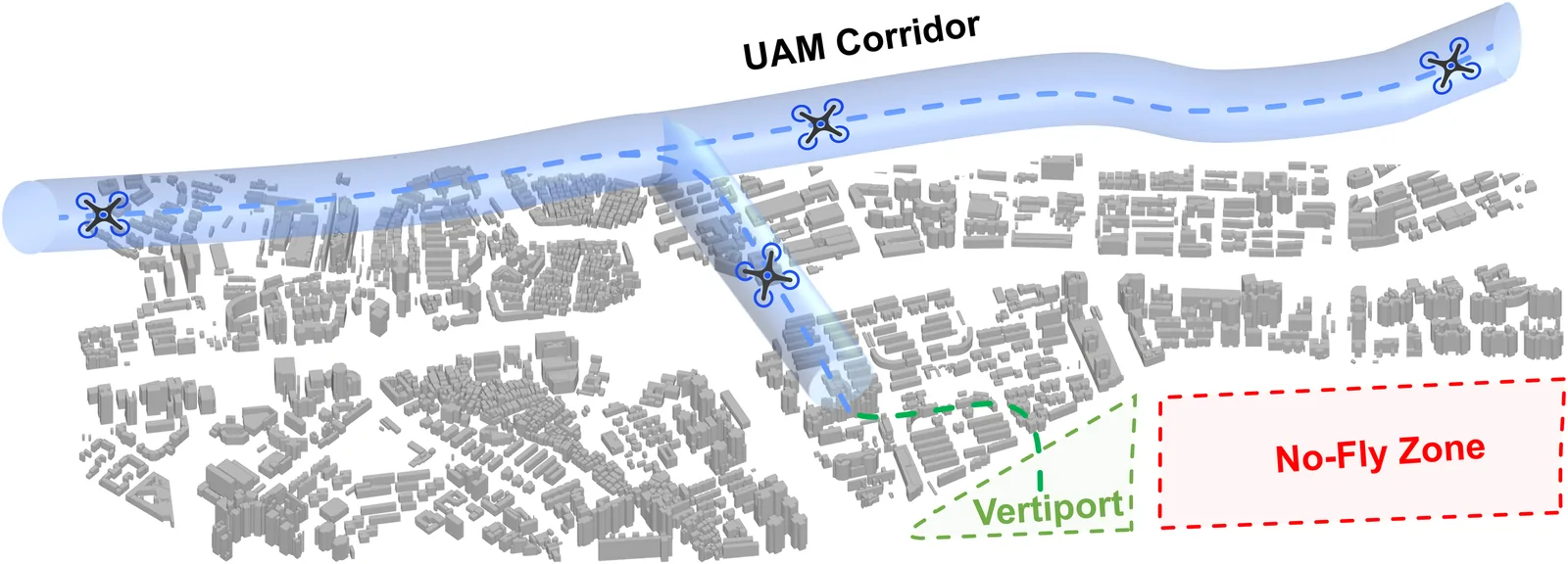
Author-generated schematic of corridor-based airspace. Building geometry is shown for illustration; no proprietary map or satellite basemap is used.

Urban environments are highly complex, with substantial variations in building density, height, and openness. In urban wind studies, CFD has been used to examine how building configurations shape connected low-resistance passages and ventilation corridors [15,16]. This study draws only a mathematical and topological analogy from that work: an obstacle-constrained vector field is used to reveal continuous planar passages and to guide smooth centerline extraction. The simulated field is synthetic and is not intended to reproduce atmospheric wind conditions, UAV demand generation, or real traffic-flow dynamics. Accordingly, the identified streamlines are interpreted as candidate spatial structures for strategic corridor planning rather than as predictions of physical UAV trajectories.

The remainder of this paper is organized as follows. Section 2 reviews related studies on the design of urban low-altitude traffic corridors (ULATCs). Section 3 introduces the proposed corridor-identification and path-evaluation methods. Section 4 presents the flow-field, corridor-network, baseline-comparison, sensitivity, and path-planning results. Section 5 discusses interpretation, applicability, and limitations, and Section 6 summarizes the main conclusions.

## 2. Related work

Structured drone route networks have been deployed in several Chinese cities, including Shenzhen and Hangzhou, to support commercial drone delivery services [17]. Zhai et al. introduced the Global Three-Dimensional Split-Tessellation Theory (GeoSOT-3D) to discretize continuous airspace into regular three-dimensional grid cells [18]. Drawing inspiration from terrestrial highway classification systems, they further designed a hierarchical route network composed of four distinct grid scales. Slama et al. proposed a low-risk urban corridor generation approach by performing risk-minimized path planning for large-scale origin–destination (OD) tasks, clustering path points, and extracting high-density, low-risk frequent passage zones [19].

Stuive and Gzara projected a three-dimensional road network into the air to construct a drone air traffic network and evaluated its performance using a constrained system optimization (CSO)–based traffic assignment model [20]. However, the resulting aerial network primarily focused on airspace above roadways, thereby failing to fully exploit available airspace resources such as parks and areas above low-rise buildings. He et al. developed a corridor-based route network planning method based on priority decoupling strategies and spatial cost functions, and subsequently proposed a network optimization approach incorporating congestion pricing [21,22]. However, this approach relies heavily on predefined OD pair topologies, which may necessitate global network replanning as the number of urban OD points increases.

Research integrating CFD with transportation has been extensively explored [23,24]. Early pioneering work by Lighthill and Whitham [25] drew an analogy between traffic flow and fluid flow. Li applied the conservation laws of fluid dynamics to traffic flow modeling and optimization [26]. Dong et al. further analyzed the similarities between traffic flow and fluid flow [27]. They constructed a two-dimensional fluid domain representing a traffic roundabout and employed the Fluent simulation platform to analyze flow smoothness, thereby identifying the underlying causes of roundabout traffic congestion. Based on these analyses, they optimized the traffic roundabout by adjusting vehicle entry angles and regulating flow velocity.

In urban wind environment research, CFD is widely employed for wind field simulation and the identification of urban ventilation corridors. Liu et al. applied CFD to simulate the urban ventilation environment of Shanghai, China, and identified 14 primary ventilation corridors using the Lowest Cost Path (LCP) method [16]. Their results demonstrated that urban building coverage and floor area ratio significantly affect wind fields, leading to variations in wind speed and direction. Lau et al. combined CFD with urban roughness modeling to simulate the urban ventilation environment in Taiwan City [15]. They further employed the A* search algorithm to identify urban ventilation corridors.

Existing studies have investigated the application of CFD to low-altitude airspace corridor design. By solving Laplace partial differential equations to derive streamlines, these studies generate flight corridors that bypass buildings, enabling drones to naturally avoid urban obstacles [12,28]. However, these methods primarily emphasize the geometric feasibility of streamlines, with limited assessment of the linear smoothness of the resulting corridors. Moreover, naturally occurring streamlines within flow fields often exhibit substantial interlacing, which increases corridor structural complexity and complicates low-altitude airspace traffic management.

To address these limitations, this paper integrates a CFD-inspired synthetic-field formulation with urban drone corridor-network planning. The proposed method uses the resulting obstacle-constrained field to identify continuous candidate passages with comparatively simple geometry under the adopted two-dimensional representation. It therefore provides a technical framework for strategic candidate-network extraction, rather than for automatic operational deployment.

## 3. Methodology

### 3.1. Theoretical framework and model formulation

This study uses CFD as a spatial structural analysis tool to identify potential ULATCs. The governing equations generate a smooth synthetic vector field whose geometry is constrained by the planar obstacle topology. Streamlines extracted from this field provide candidate obstacle-avoiding centerlines for a structured aerial network. The continuum representation is therefore a computational device for passage extraction; it does not assert that UAV traffic behaves physically as an incompressible fluid.

Given the computational overhead associated with three-dimensional simulation in high-density urban domains, this research adopts a two-dimensional formulation to screen the dominant planar connectivity of a candidate corridor network over a 5×5 km^2^ study area. This representation preserves connectivity only under the adopted rasterized obstacle mask; it does not establish equivalence with the full three-dimensional airspace. The output should therefore be treated as a strategic-level candidate backbone that requires subsequent three-dimensional obstacle and operational safety assessment before implementation.

### 3.2. Urban environment and airspace modeling

To evaluate the efficacy of the proposed methodology, the Futian District in Shenzhen, China, was selected as the empirical study site. As a global pioneer in the low-altitude economy, Shenzhen hosts intensive drone operations from major logistics providers such as Meituan and Fengyi. However, the current fragmented route planning paradigm necessitates a unified corridor network to serve as a standardized airspace base map and mitigate coordination complexities. In alignment with the “National Airspace Basic Classification Method” (2023) by the Civil Aviation Administration of China (CAAC) [29], this study focuses on Class W airspace (below 120 m AGL), which constitutes the primary operational domain for low-altitude logistics UAVs.

The study area encompasses a 5×5 km^2^ zone within the central urban district, incorporating eight designated no-fly zones above major parks and government facilities, as illustrated in Fig 2. Despite the high density of commercial structures, approximately 41% of buildings in this region are lower than 30 m. Based on China’s logistics route specifications, which stipulate operational altitudes between 40 m and 120 m, the strategic screening considers corridors in the 90–120 m layer above zones where mapped buildings are lower than 30 m. Structures exceeding this threshold are treated as rigid obstacles in the planar mask. Under these static building-height assumptions, the 30 m threshold provides a nominal vertical separation of at least 60 m from the lower boundary of the selected layer; it is not an operational safety guarantee.

**Fig 2:**
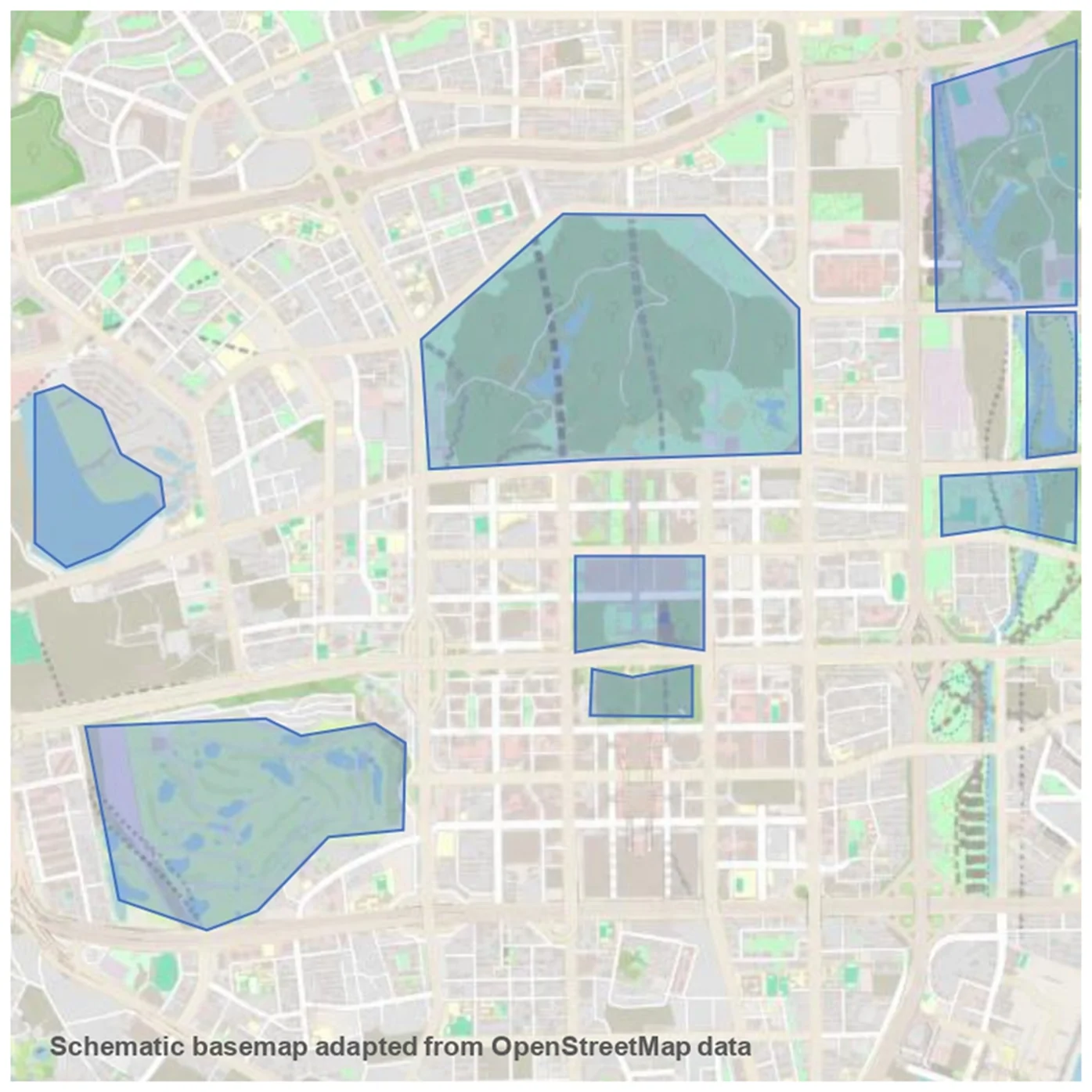
Study area and modeled constraints. The designated no-fly-zone polygons were compiled by the authors. The background is a schematic basemap adapted by the authors from OpenStreetMap data and is used for contextual visualization only.

China’s “Specifications for Designating Urban Scenario Light and Small Unmanned Aerial System Logistics Routes” stipulate that designated flight routes should be above 40 m in altitude, with a maximum height not exceeding 120 m. The establishment of ULATCs is typically planned within an altitude range of 90–120 m. Accordingly, this study models buildings higher than 30 m and designated prohibited airspace as obstacles. The available input does not comprehensively represent thin or temporary vertical objects such as cranes, tower cranes, antennas, masts, power lines, and construction equipment. These objects must be incorporated through higher-resolution three-dimensional surveys and operational constraint layers before any candidate corridor is used in practice.

This study primarily uses the 3D-GloBFP dataset to obtain urban building outlines and height data [30]. The building-height estimation model in this dataset achieves an *R*^2^ ranging from 0.66 to 0.96, with a root mean square error (RMSE) between 1.9 and 14.6 m. These data are used for strategic-level screening, but their uncertainty and spatial resolution do not replace detailed obstacle surveys or site-specific safety assessment. Buildings near the 30 m classification threshold and locally tall or narrow objects require particular verification.

For environmental modeling, a 50 m × 50 m two-dimensional grid is used for discretization. Navigable and non-navigable regions are generated through obstacle mapping. First, the urban building-outline and height data are mapped onto the grid, as shown in Fig 3A. A navigable cell represents planar availability at the adopted screening resolution and should not be interpreted as certifying a 50 m-wide operational corridor. The X and Y axes are expressed in UTM Zone 50N coordinates (meters), maintaining consistency with the GIS inputs and subsequent spatial calculations. Cells containing mapped buildings higher than 30 m are designated as non-navigable obstacles. The resulting obstacle map is shown in Fig 3B.

**Fig 3:**
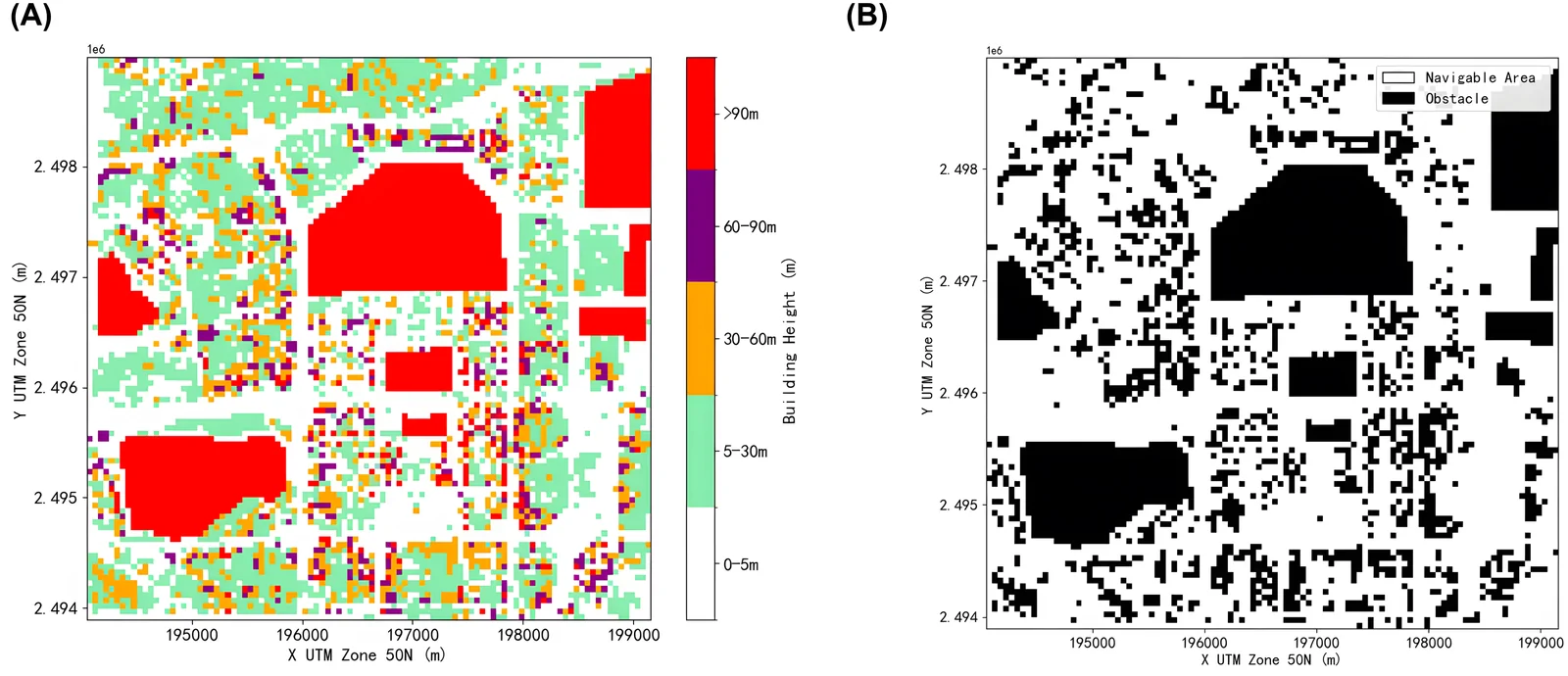
Author-generated discretized building-height and obstacle maps derived from the 3D-GloBFP data [30] and the designated no-fly-zone polygons. (A) Basic discrete map. (B) Obstacle map.

### 3.3. CFD simulation

This study employs PhiFlow 3.4.0 with the PyTorch 2.5.1 CUDA 12.1 backend to construct the synthetic velocity fields [31]. PhiFlow provides a Python-based interface for solving partial differential equations and representing spatial fields. The overall workflow is illustrated in Fig 4 and summarized in Algorithm 1.

**Fig 4:**
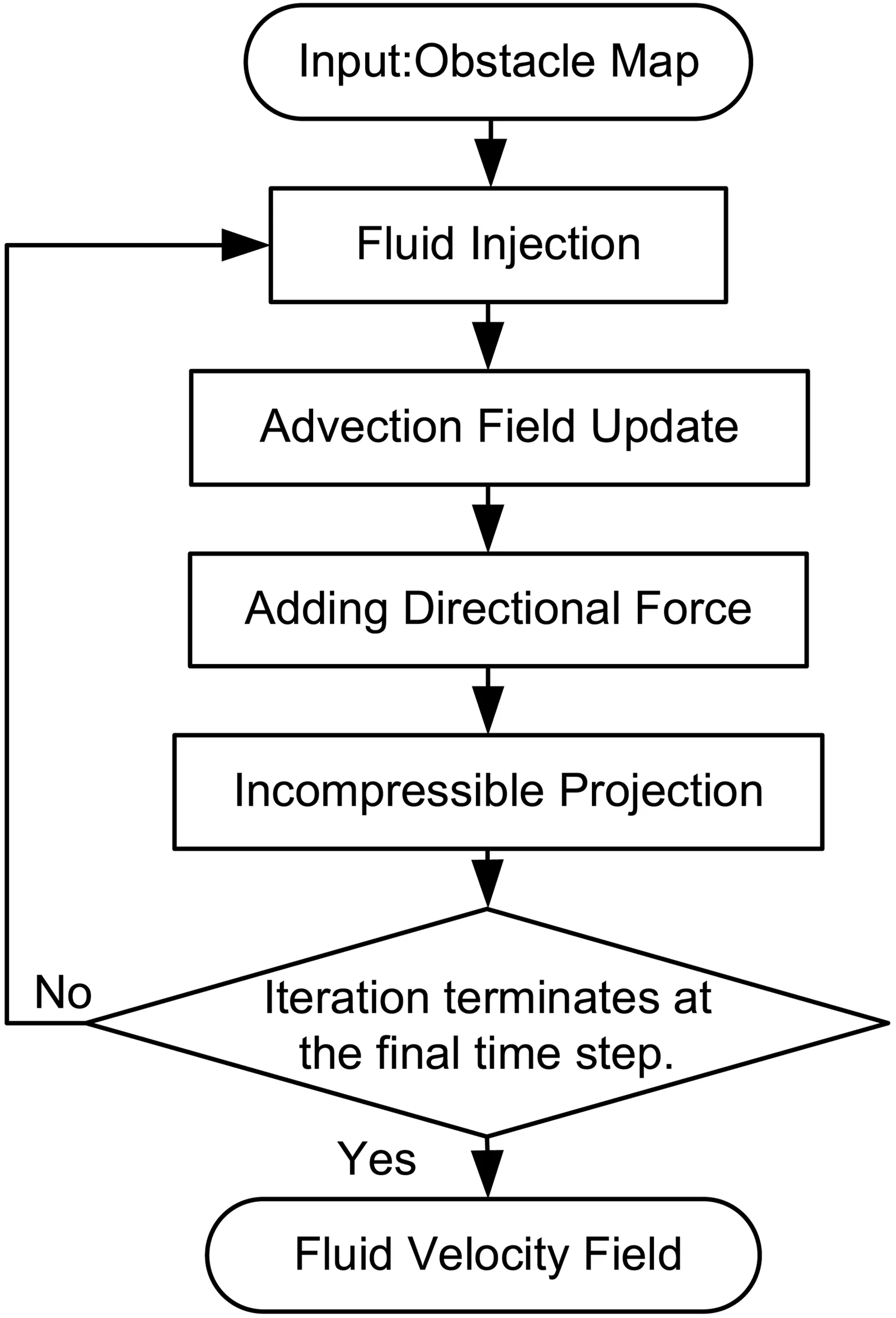
CFD simulation process.

**Algorithm 1 Urban CFD simulation algorithm**

**Require:** Discrete urban obstacle map

**Ensure:** Velocity fields $\left\{V_{1},V_{2},...,V_{N}\right\}$ under multiple inflow directions

1: Identify enclosed regions in the obstacle map and correct them as solid obstacles

2: Construct solid boundary geometry within the simulation domain

3: Obtain urban obstacle map for CFD simulation

4: Define inflow direction set Directions={South,North,East,West}

5: **for all** d∈Directions **do**

6:      Initialize density field Density←0

7:      Initialize velocity field Velocity←0

8:      Initialize pressure field Pressure←0

9:      Configure inflow condition *Inflow* and directional driving force *ForceVector* according to direction *d*

10:      **for** *t* = 1 to *T* **do** ▷ *T* is the predefined number of simulation time steps

11:        Density←Advect(Density,Velocity,Δt)+Inflow     ▷ Density field advection

12:        Velocity←Advect(Velocity,Velocity,Δt)         ▷ Velocity field advection

13:        Velocity←Velocity+Density×ForceVector       ▷ Apply directional driving force

14:        Velocity←Velocity×FreeSpaceMask         ▷ Obstacle constraint to prevent flow penetration

15:        (Velocity,Pressure)←ProjectIncompressible(Velocity,Geometry,Pressure)   ▷ Enforce incompressibility

16:   **end for**

17:   $V_{d}\leftarrow Velocity$              ▷ Output velocity field for direction *d*

18: **end for**

19: **return** $\left\{V_{1},V_{2},...,V_{N}\right\}$

#### 3.3.1. Urban environment processing and modeling.

Based on the urban binary obstacle mask constructed in Section 3.2, the mask is converted into a geometry representation compatible with PhiFlow. A flood-fill algorithm is employed to detect the presence of enclosed regions within the computational domain [32]. If enclosed regions are identified, they are filled to prevent numerical instability during pressure field computation.

#### 3.3.2. Multi-directional steady-state fluid injection.

To comprehensively identify the urban traffic corridor network, steady-state flow is injected from the east, west, south, and north boundaries of the simulation domain. These four simulations are conducted independently to capture traffic corridors under different dominant flow directions.

#### 3.3.3. Computational fluid dynamics numerical solution.

The core of the simulation is based on PhiFlow’s numerical solution of the incompressible Navier–Stokes equations, as given in Eq (1):


$$\begin{array}{c} \frac{\partial 𝐯}{\partial t}+(𝐯\cdot \nabla )𝐯=-\frac{1}{\rho }\nabla p+\nu \nabla^{2}𝐯+𝐟 \end{array}$$ (1)

Where v denotes the velocity field, ρ is the fluid density, *p* is the pressure, ν is the kinematic viscosity, and f denotes the external body force.

**Advection field update:** In fluid simulation, advection describes the process by which physical quantities, such as density and velocity, are transported and updated by the fluid motion. The governing equation for this process is given in Eq (2).


$$\begin{array}{c} \frac{\partial \phi }{\partial t}+𝐮\cdot \nabla \phi =0 \end{array}$$ (2)

Where ϕ represents the physical quantity being advected (primarily fluid velocity and density in this paper), *t* is time, 𝐮 is the velocity vec*t*or field of the fluid, and ∇ϕ is the gradient of the physical quantity ϕ. The term 𝐮·∇ϕ represents the convective component, describing the transport of ϕ along the direction of the velocity field 𝐮.

In this study, the MacCormack algorithm is employed to compute the advected fluid density field [33]. The MacCormack method is a second-order accurate predictor–corrector finite-difference scheme. This method effectively reduces numerical dissipation and preserves sharp density field boundaries by alternately applying forward and backward spatial differencing. Compared with more complex high-order schemes, such as ENO and WENO, the MacCormack scheme provides a favorable balance between accuracy and computational efficiency. The computational procedure of the MacCormack scheme is detailed in Eq (3).


$$\begin{array}{c} \left\{ \begin{array}{cc} {\tilde{\phi }}_{i,j} & =\phi_{i,j}^{n}-\Delta t\left(u_{i,j}^{n}\frac{\phi_{i+1,j}^{n}-\phi_{i,j}^{n}}{\Delta x}+v_{i,j}^{n}\frac{\phi_{i,j+1}^{n}-\phi_{i,j}^{n}}{\Delta y}\right)\\ \phi_{i,j}^{n+1,\text{temp}} & =\phi_{i,j}^{n}-\Delta t\left(u_{i,j}^{n}\frac{{\tilde{\phi }}_{i,j}-{\tilde{\phi }}_{i-1,j}}{\Delta x}+v_{i,j}^{n}\frac{{\tilde{\phi }}_{i,j}-{\tilde{\phi }}_{i,j-1}}{\Delta y}\right)\\ \phi_{i,j}^{n+1} & =\frac{1}{2}(\phi_{i,j}^{n}+\phi_{i,j}^{n+1,\text{temp}}) \end{array}\right. \end{array}$$ (3)

Where $\phi_{i,j}^{n}$ represents the physical quantity value at grid point (*i*,*j*) and time step *n*; $u_{i,j}^{n}$ and $v_{i,j}^{n}$ are the velocity components in the *x* and *y* directions at grid point (*i*,*j*) and time step *n*; Δt is the time step size; Δx and Δy are the spatial grid step sizes in the *x* and *y* directions; $\phi_{i+1,j}^{n}$ and $\phi_{i,j+1}^{n}$ represent physical quantity values at adjacent grid points at time step *n*; $\tilde{\phi }i,j$ is the temporary predicted value at the next time step *n* + 1 for ϕi,j, computed using the forward differencing method; $\phi_{i,j}^{n+1,\text{temp}}$ is another temporary value obtained by correcting ϕi,j using backward spatial differencing based on the predicted value $\tilde{\phi }i,j$.and $\phi_{i,j}^{n+1}$ is the final updated value at grid point (*i*,*j*) and time step *n* + 1.

The semi-Lagrangian algorithm is employed to compute the advected fluid velocity field [34]. As a streamline-based advection scheme, the semi-Lagrangian method performs interpolation and reconstruction of physical quantities by tracing streamlines backward along the velocity field. Owing to its unconditional stability, this method maintains numerical stability even with large time steps, without a significant increase in computational cost. Compared with the CFL constraints of traditional Eulerian methods, the semi-Lagrangian approach provides a stable numerical representation of the obstacle-constrained synthetic velocity patterns used in this study. The computational procedure of the semi-Lagrangian method is presented in Eq (4).


$$\begin{array}{c} \left\{ \begin{array}{cc} {𝐱}_{p} & =𝐱-𝐮(𝐱,t^{n})\Delta t\\ \phi^{n+1}(𝐱) & =\text{Interpolate}(\phi^{n},{𝐱}_{p}) \end{array}\right. \end{array}$$ (4)

Where 𝐱 is the coordinate of the current grid point; ${𝐱}_{p}$ is the estimated starting position of the fluid particle at the previous time step $t^{n}$ computed using backward tracing; $𝐮(𝐱,t^{n})$ is the velocity vector at the current grid point 𝐱 and previous time step $t^{n}$; Δt is the time step size; $\phi^{n+1}(𝐱)$ is the physical quantity value (velocity) at the current grid point 𝐱 and new time step $t^{n+1}$; and $\text{Interpolate}(\phi^{n},{𝐱}_{p})$ represents the interpolation of the physical quantity ϕ at the previous time step $t^{n}$ evaluated at the backtracked point ${𝐱}_{p}$, typically using bilinear or higher-order interpolation methods.

**Addition of directional driving force:** A directional driving force is applied according to the density field to orient the synthetic flow toward a prescribed target boundary. Regions with higher density receive larger velocity increments. This numerical construction promotes continuous directional passages in the obstacle-constrained field; it does not model UAV acceleration, pilot behavior, or traffic demand.

**Incompressible projection:** At each time step, a predicted velocity field is first computed through advection and the application of directional forces. The resulting velocity field may not satisfy the incompressibility constraint, that is, its divergence may be non-zero. To enforce incompressibility, a corrective pressure is obtained by solving a pressure Poisson equation, and the resulting pressure gradient is used to update the velocity field. This numerical operation produces a smooth divergence-free field around the modeled obstacles; it is used as a geometric passage indicator and does not emulate an onboard UAV obstacle-avoidance controller. The computational procedure for the Incompressible projection is presented in Eq (5).


$$\begin{array}{c} \left\{ \begin{array}{cc} {𝐮}^{n+1} & ={𝐮}^{*}-\frac{\Delta t}{\rho }\nabla p^{n+1}\\ \nabla \cdot {𝐮}^{n+1} & =0\\ \nabla^{2}p^{n+1} & =\frac{\rho }{\Delta t}\nabla \cdot {𝐮}^{*} \end{array}\right. \end{array}$$ (5)

Where ${𝐮}^{n+1}$ is the corrected velocity field at time step *n* + 1 satisfying the incompressibility condition; ${𝐮}^{*}$ is the predicted velocity field obtained after advection and external forces (typically with non-zero divergence); $p^{n+1}$ is the pressure field to be solved at time step *n* + 1; ρ is the fluid density; Δt is the time step size; $\nabla p^{n+1}$ is the pressure gradient representing the correction effect of pressure differences on velocity; $\nabla \cdot {𝐮}^{n+1}=0$ represents the mass conservation requirement for incompressible fluids; and $\nabla^{2}p^{n+1}$ is the Laplace operator of the pressure field, describing the spatial curvature of pressure.

After the prescribed time-step iterations, the resulting field reveals regions with relatively high synthetic velocity magnitude and consistent direction under each imposed boundary condition. The four directional fields are stored for subsequent streamline tracing. Their magnitudes are interpreted only on a relative, within-simulation basis and are not calibrated atmospheric wind speeds.

### 3.4. Corridor identification

Traditional path-finding algorithms typically rely on predefined OD pairs; however, such pairs may not be available during strategic corridor-network screening. The proposed velocity-field-based method instead traces relatively high-magnitude, directionally consistent regions in the synthetic fields described in Section 3.3. It therefore identifies candidate passages without requiring predefined OD pairs. The overall workflow is illustrated in Fig 5.

**Fig 5:**
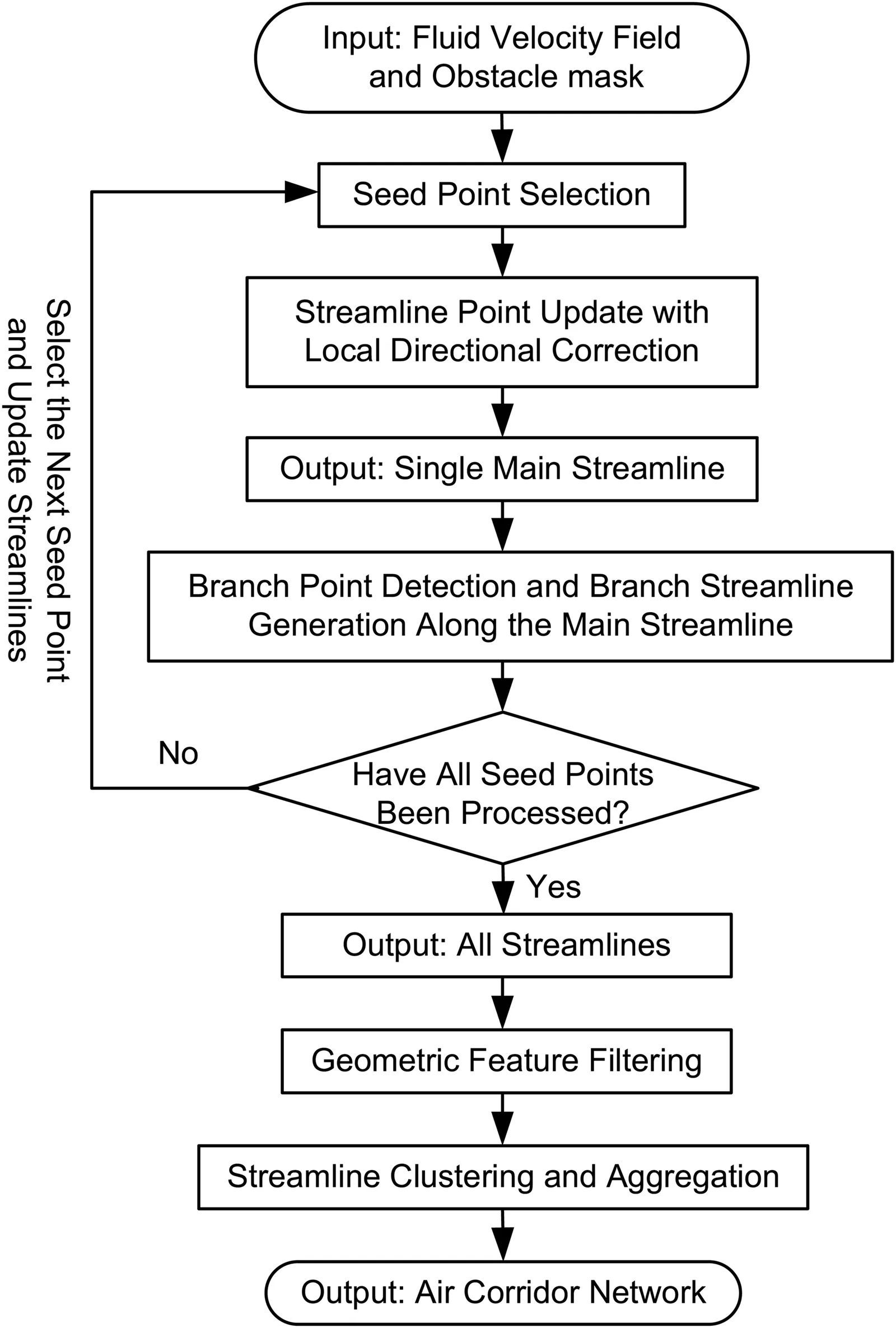
Corridor identification process.

#### 3.4.1. Streamline tracing algorithm.

In fluid mechanics, a streamline is tangent to the local velocity vector. Here, the same mathematical construction is used to extract candidate geometric centerlines from a synthetic field, rather than to predict individual UAV trajectories. The Flow Field-Driven High-Velocity Corridor Tracing Algorithm (FHVCT) traces relatively high-magnitude passages and applies obstacle-aware redirection and branching. Its pseudocode is provided in Algorithm 2.

**Algorithm 2 Streamline tracing (Flow field-driven)**

**Require:** Directional velocity fields $\left\{V_{1},V_{2},...,V_{N}\right\}$, obstacle map *O*

**Ensure:** Streamline set ℒ

1: Define inflow direction set Directions={South,North,East,West}

2: Initialize streamline set ℒ←∅

3: **for all** d∈Directions **do**

4:   Select corresponding velocity field ${𝑉}_{d}$

5:   Generate seed points set $𝒮=\{s_{1},s_{2},...,s_{n}\}$

6:   **for all** $s_{i}\in 𝒮$ **do**

7:    Initialize main streamline $\ell_{\text{main}}$ starting from $s_{i}$

8:    **while** streamline termination conditions are not met **do**

9:     **if** current point intersects obstacle or enters low-velocity region **then**

10:     Apply path redirection

11:    **end if**

12:    Update streamline point using RK4 integration

13:   **end while**

14:   Save main streamline $\ell_{\text{main}}$ and add it to ℒ

15:   Detect bifurcation points $\left\{b_{1},b_{2},...,b_{m}\right\}$ along $\ell_{\text{main}}$

16:   **for all** $b_{j}\in \{b_{1},b_{2},...,b_{m}\}$ **do**

17:    Extract prefix streamline $\ell_{\text{prefix}}$ of $\ell_{\text{main}}$ up to $b_{j}$

18:    Initialize branch streamline $\ell_{\text{tail}}$ from $b_{j}$

19:    Trace $\ell_{\text{tail}}$ using the same RK4-based procedure with redirection

20:    Construct branch streamline $\ell_{\text{branch}}\leftarrow \ell_{\text{prefix}}\cup \ell_{\text{tail}}$

21:    Add $\ell_{\text{branch}}$ to ℒ

22:   **end for**

23:  **end for**

24: **end for**

25: **return** ℒ

**Starting Point Selection:** After completion of the CFD simulations, a velocity threshold is applied to each directional velocity field to prevent streamline tracing from entering low-velocity regions. Streamline tracing seed points are uniformly sampled along the four inlet boundaries of the simulation domain, ensuring that the resulting streamlines comprehensively cover potential traffic regions.

**Streamline point update:** In the streamline tracing process, the fourth-order Runge–Kutta (RK4) method is employed for kinematic integration to accurately simulate particle trajectories [35]. Compared with the first-order Euler and second-order RK2 methods, RK4 maintains high integration accuracy under rapidly varying velocity fields, thereby preventing streamlines from erroneously intersecting obstacles or exhibiting spurious oscillations. A streamline is defined as a trajectory that is everywhere tangent to the local velocity vector. The RK4 scheme enhances integration accuracy by evaluating the velocity field at multiple intermediate points, enabling streamline points to be updated more precisely along the local flow direction. The corresponding formulation is given in Eq (6).


$$\begin{array}{c} \left\{ \begin{array}{cc} {𝐤}_{1} & =\Delta t\cdot 𝐕(x_{n},y_{n})\\ {𝐤}_{2} & =\Delta t\cdot 𝐕(x_{n}+0.5{𝐤}_{1x},\;y_{n}+0.5{𝐤}_{1y})\\ {𝐤}_{3} & =\Delta t\cdot 𝐕(x_{n}+0.5{𝐤}_{2x},\;y_{n}+0.5{𝐤}_{2y})\\ {𝐤}_{4} & =\Delta t\cdot 𝐕(x_{n}+{𝐤}_{3x},\;y_{n}+{𝐤}_{3y})\\ \left(x_{n+1},y_{n+1}\right) & =(x_{n},y_{n})+\frac{1}{6}({𝐤}_{1}+2{𝐤}_{2}+2{𝐤}_{3}+{𝐤}_{4}) \end{array}\right. \end{array}$$ (6)

Where 𝐕(x,y)=(U(x,y),V(x,y)) is the fluid velocity vector at point (*x*, *y*), and Δt is the integration time step.

**Streamline obstacle avoidance and path redirection:** This numerical procedure redirects a candidate centerline when it approaches a modeled obstacle or enters a low-relative-velocity region, as illustrated in Fig 6A. The light-gray regions denote obstacles, while blue and red indicate lower and higher relative synthetic velocity, respectively. Within a predefined search radius, the algorithm evaluates neighboring candidate points and selects the next point using the scoring function in Eq (7). This operation is part of offline corridor extraction and should not be interpreted as a real-time UAV control model.


$$\begin{array}{c} \text{Score}=w_{\text{speed}}\cdot \left(\frac{V_{\text{sampled}}}{V_{\text{max}}}\right)+w_{\text{mask}}\cdot M_{\text{sampled}}+w_{\text{angle}}\cdot \cos\theta \end{array}$$ (7)

where *V*_sampled_ is the flow velocity at the sampling point, *V*_max_ is the maximum flow velocity in the search area, *M*_sampled_ is the mask value at the sampling point, and cosθ represents the directional consistency between the current streamline direction and the direction toward the sampling point. The weights *w*_speed_, *w*_mask_, and *w*_angle_ control the relative contributions of these three factors. The parameter values used for streamline-based obstacle avoidance and path redirection are summarized in Table 1.

**Fig 6:**
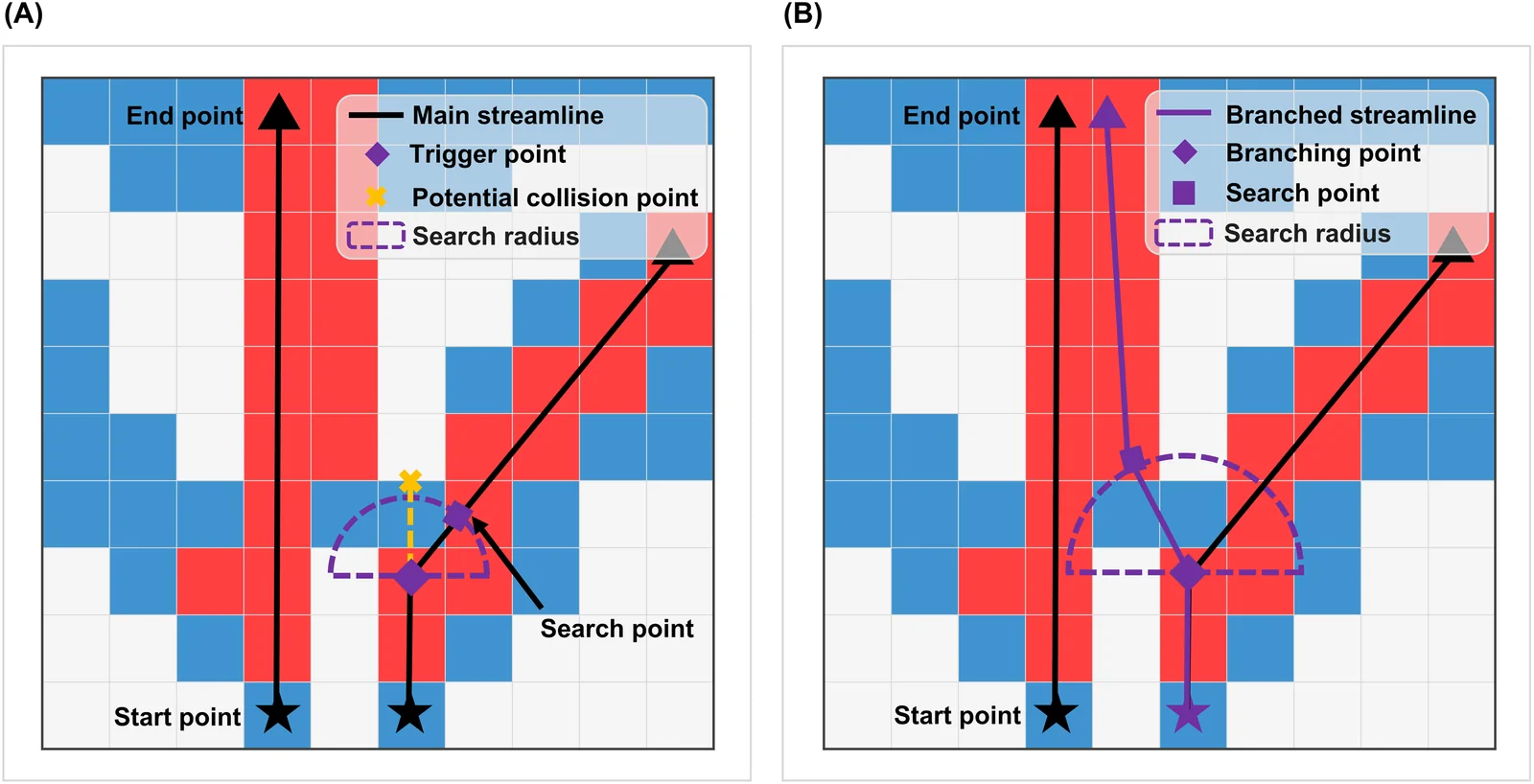
Illustration of streamline tracing. (A) Streamline path redirection. (B) Bifurcating streamline generation.

**Table 1:** Parameters for streamline-based obstacle avoidance and path redirection.

Parameter	Value	Description
Velocity weight in scoring function	0.4	Encourages sampled points to move along directions with higher flow velocity.
Obstacle distance weight in scoring function	0.2	Rewards sampled points that are farther away from obstacles.
Search angle weight in scoring function	0.4	Ensures path smoothness by penalizing abrupt directional changes.
Obstacle proximity threshold	0.75	When the mask value falls below this threshold, the streamline is considered to be approaching an obstacle, triggering obstacle avoidance.
Low-velocity threshold	30%	Defined as the 30th percentile of the effective velocity over the entire domain; regions below this value are treated as low-velocity areas.
Maximum allowable turning angle	90°	Constraint imposed to maintain path smoothness.

**Bifurcating streamline generation**. To enhance the connectivity and coverage of the traced streamlines, a bifurcation mechanism is introduced during the tracing process, as illustrated in Fig 6B. When a main streamline passes through a region and discovers other potential high-velocity regions nearby with a small difference in direction, the algorithm generates a new bifurcating streamline at this point. It then smoothly connects the main streamline and the starting point of the bifurcating streamline using a Bezier curve to ensure path continuity and naturalness. The parameters and corresponding descriptions for bifurcating streamline generation are summarized in Table 2.

**Table 2:** Parameters for bifurcating streamline generation.

Parameter	Value	Description
Bifurcation trigger condition	≥1.5	A bifurcation is triggered when the maximum surrounding velocity exceeds 1.5 times the velocity at the current streamline point.
Maximum number ofbifurcations	30	The maximum number of bifurcating streamlines allowed to be generated from a single main streamline.
Maximum bifurcation angle	90°	The maximum allowable angular deviation between the direction of a bifurcating streamline and the main streamline.
Bifurcation sampling radius	100 m	The radial distance centered at the current point within which new candidate paths are explored.
Minimum bifurcation length	500 m	Bifurcating streamlines shorter than this length are discarded to ensure path effectiveness.
Number of Bézier control points	8	The number of control points used in the Bézier curve for smoothly connecting the main streamline and the bifurcating streamline.

The resulting streamlines are candidate centerlines that avoid obstacles represented in the two-dimensional mask. They are not certified flight corridors and require the subsequent filtering, redundancy removal, three-dimensional verification, and operational assessment described below.

#### 3.4.2. Corridor extraction and optimization.

The streamline tracing algorithm produces a large number of streamlines, many of which are redundant due to insufficient length or low straightness. To extract high-quality traffic corridors, this study adopts a two-stage processing pipeline consisting of feature filtering followed by redundancy removal. The pseudocode of the proposed algorithm is presented in Algorithm 3.

**Algorithm 3 Streamline post-processing**

**Require:** All streamline ℒ

**Ensure:** Air corridor network ${𝒩}_{corridor}$

1:                         ▷ **Geometric filtering**

2: **for all** streamline ℓ∈ℒ **do**

3:   Compute geometric features of ℓ (length, straightness)

4:   **if** ℓ does not satisfy predefined criteria **then**

5:    Remove ℓ from ℒ

6:   **end if**

7: **end for**

8:                     ▷ **Initial deduplication (Clustering)**

9:  Cluster the set ℒ into *k* clusters: $\left\{{𝒞}_{1},{𝒞}_{2},...,{𝒞}_{k}\right\}$

10: Initialize representative streamline set $L_{rep}\leftarrow \emptyset$

11: **for all** cluster ${𝒞}_{i}\in \{{𝒞}_{1},...,{𝒞}_{k}\}$ **do**

12:   Compute a quality score for each streamline $\ell \in {𝒞}_{i}$

13:   Select representative streamline $\ell_{i}^{*}$ with the highest score from ${𝒞}_{i}$

14:   Add $\ell_{i}^{*}$ to ${ℒ}_{rep}$

15: **end for**

▷ **Cross-Cluster Refinement**

16: **for all** each distinct pair $\left(\ell_{i}^{*},\ell_{j}^{*}\right)$ from ${ℒ}_{rep}$ **do**

17:   Segment $\ell_{i}^{*}$ and $\ell_{j}^{*}$ into local sub-segments

18:   Perform local clustering on the collected sub-segments

19:   **if** local redundancy (high similarity) is detected **then**

20:    Remove redundant sub-segments from the lower-score streamline

21:   **end if**

22: **end for**

23: ${𝒩}_{corridor}\leftarrow {ℒ}_{rep}$

24: **return** ${𝒩}_{corridor}$

First, paths that are short or not smooth are excluded based on streamline length and straightness. The straightness metric is computed as defined in Eq (8):


$$\begin{array}{c} \text{S}=\frac{\text{D}}{\text{L}} \end{array}$$ (8)

Where D is the direct distance between the path’s start and end points, and L is the path’s total length.

This study selects streamlines with lengths exceeding 2500 m and straightness values greater than 0.75 as preliminary corridor candidates. However, it is necessary to retain a unique and highest-quality representative path among geometrically similar candidates, as illustrated in Fig 7A, where black regions denote obstacles. To achieve this, the DBSCAN clustering algorithm is employed to group geometrically similar paths [36]. DBSCAN exhibits notable advantages for spatial trajectory clustering, including the absence of a requirement for a predefined number of clusters, the ability to identify clusters of arbitrary shapes, and strong robustness to noisy streamlines. Consequently, it is more suitable than K-means or spectral clustering for complex path sets with unknown cardinality. Each candidate corridor is subsequently subjected to a comprehensive quality evaluation. The scoring system integrates multiple metrics, including corridor length, straightness, path curvature, and average flow velocity. These metrics are combined through weighted summation to produce a normalized quality score ranging from 0 to 1. The parameters and corresponding descriptions used in the initial deduplication process are summarized in Table 3.

**Table 3:** Parameters for initial clustering and deduplication.

Parameter	Value	Description
Clustering criterion	overlap	Similarity between corridors is determined based on the degree of overlap between their buffer zones.
Similarity buffer radius	250 m	Radius of the buffer zone used to compute corridor overlap.
Clustering similarity threshold	0.6	Corridors with an overlap ratio exceeding this threshold are assigned to the same cluster.
Length score weight	0.22	Weight of the total corridor length, normalized with 5000 m as the reference value.
Straightness score weight	0.25	Weight assigned to the corridor straightness metric.
Smoothness score weight	0.33	Weight associated with path smoothness.
Average velocity score weight	0.20	Weight of the average corridor flow velocity, normalized using 70% of the maximum velocity as the reference.

**Fig 7:**
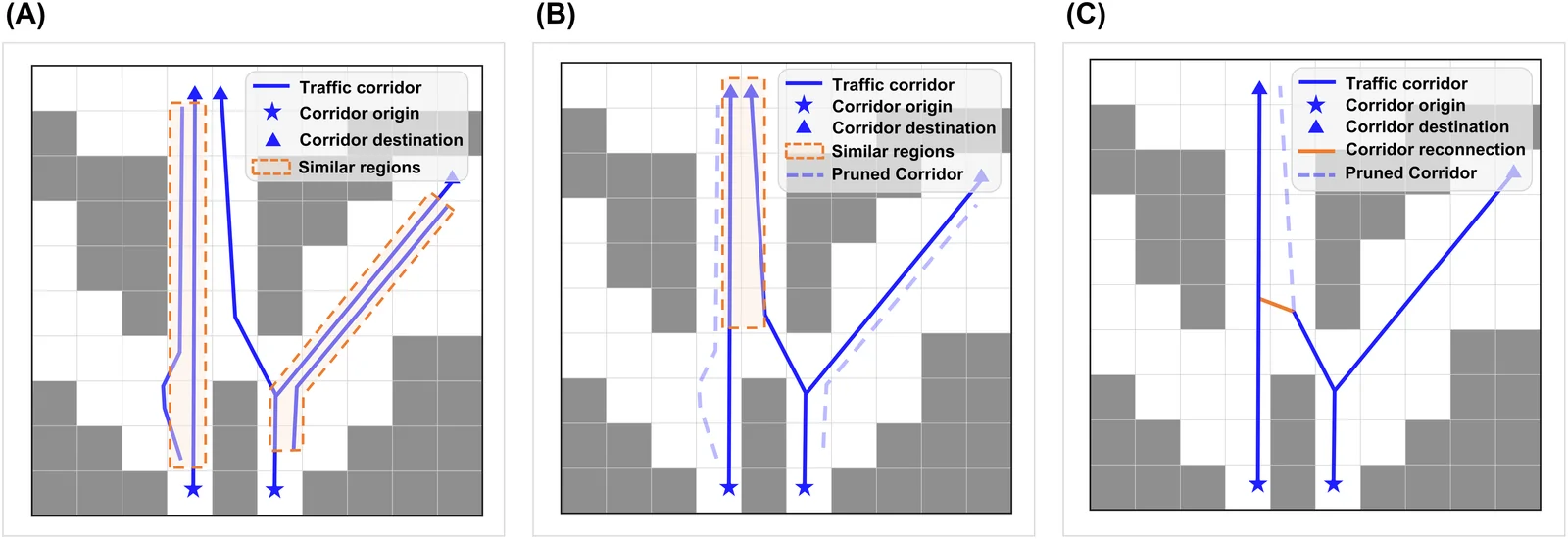
Illustration of corridor extraction and optimization. (A) Initial deduplication. (B) Cross-cluster refinement. (C) Final corridor network.

The candidates from the east-, west-, south-, and north-driven fields are pooled before clustering. No inflow direction is assigned priority. Pairwise similarity is evaluated from buffered geometric overlap without regard to digitized line orientation; consequently, geometrically coincident paths traced in opposite directions are treated as candidates for the same physical corridor. Candidate pairs whose overlap exceeds the specified threshold form the same density-connected cluster, and only the highest-quality representative is retained. Thus, a segment identified under multiple inflow conditions is counted once after primary clustering. Partial overlaps that do not meet the whole-path clustering criterion are handled in the subsequent local cross-cluster refinement. The source direction remains an intermediate provenance attribute but does not affect the representative-selection rule.

After the initial deduplication stage, local overlaps may still persist among corridors belonging to different clusters. To further simplify the corridor network, a cross-cluster refinement strategy is employed. First, the preliminarily filtered corridors are segmented into fixed-length subsegments. The geometric similarity between these subsegments is then evaluated. When high similarity is detected, the overlapping portions are removed from the lower-quality corridor, as illustrated in Fig 7B. The parameters and corresponding descriptions used in the cross-cluster refinement process are summarized in Table 4. The final corridor network is obtained through this local refinement process followed by smoothing and connection, as shown in Fig 7C.

**Table 4:** Parameters for cross-cluster refinement.

Parameter Name	Value	Description
Segmentation length	50 m	Length used to segment corridors into small subsegments for comparison.
Similarity buffer radius	100 m	Buffer radius used to compute corridor overlap.
Segment similarity threshold	0.5	Subsegments with a combined similarity exceeding this threshold are regarded as overlapping.
Segment similarity metric	0.4×overlap+0.6×Hausdorff	Composite similarity score combining spatial overlap and shape similarity.
Merging trigger threshold	50 m	Pruning is triggered only when the total length of overlapping segments exceeds this threshold.

#### 3.4.3. Path planning method.

This study adopts a corridor-based segmented path-planning framework that uses a pre-identified corridor network to generate obstacle-screened routes from origin to destination. The A* algorithm is employed for path planning. The core principle of the A* algorithm lies in evaluating the total cost of each node and iteratively expanding the node with the lowest estimated cost to identify the least-cost path. The cost function of the A* algorithm is defined in Eq (9).


$$\begin{array}{c} f(n)=g(n)+h(n) \end{array}$$ (9)

where *f*(*n*) is the total estimated cost from the start node to the end node, passing through node *n*, *g*(*n*) is the actual movement cost from the start node to the current node *n*, and *h*(*n*) is the estimated heuristic cost from the current node *n* to the end node.

Each flight mission is divided into the following three stages:

**Access stage (origin-to-corridor):** The UAV departs from its origin and plans the shortest feasible path to connect to the nearest accessible low-altitude corridor node. Obstacle avoidance with respect to surrounding buildings is explicitly considered at this stage.

**Traversal stage (corridor network):** After entering the corridor network, the UAV travels along predefined corridors until reaching the corridor node closest to the target destination. Path planning at this stage relies on the pre-constructed corridor network map.

**Egress stage (corridor-to-destination):** The UAV exits the corridor network at the selected exit node and plans the shortest path to the final destination. Similar to the first stage, obstacle avoidance with respect to buildings is taken into account.

This study considers UAV flight in a two-dimensional space, simulating a scenario in which the UAV ascends vertically from the origin to a designated flight altitude, cruises at this altitude to a position above the destination, and then descends vertically. The corridor-based segmented path-planning method developed under this framework is compared with the free-flight mode using path length, average flight velocity, path curvature, and total flight time to quantify differences in route geometry and kinematic performance. The UAV velocity update accounts for the platform’s maximum velocity and acceleration constraints at each time step; when negotiating curved segments, the UAV is additionally subject to a turn-related speed constraint. These modeled constraints do not constitute a collision-risk or operational-safety assessment.

## 4. Results

### 4.1. CFD simulation results

Fig 8 presents the synthetic velocity fields for the Futian case. White areas denote modeled obstacles, primarily buildings higher than 30 m and designated prohibited airspace. For each inflow direction, the color map shows the percentile-normalized magnitude of the corresponding synthetic velocity field: dark blue indicates lower relative magnitude and red indicates higher relative magnitude. The scale is dimensionless and does not represent atmospheric wind speed or UAV speed in physical units. Higher relative magnitudes tend to occur along continuous open strips, whereas densely obstructed areas generally show lower values.

**Fig 8:**
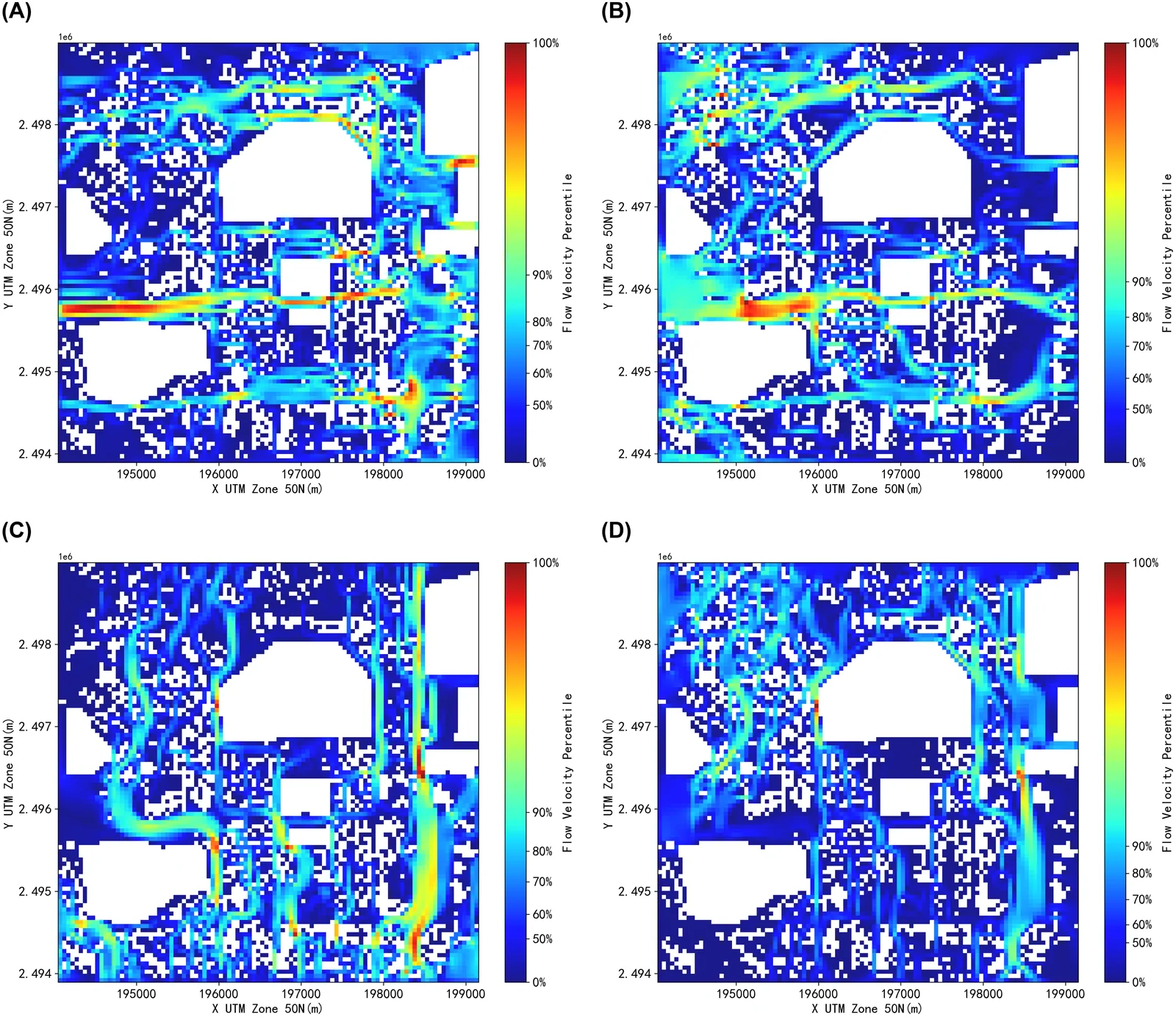
Author-generated synthetic velocity fields under four imposed inflow directions: (A) east, (B) west, (C) south, and (D) north. Colors show the within-direction percentile-normalized velocity magnitude and do not represent atmospheric wind or UAV speed in physical units.

Under the adopted mathematical formulation, relatively high-magnitude regions indicate passages that are less constrained by the modeled planar obstacles. These regions are therefore used as inputs for candidate centerline extraction. Their presence alone does not demonstrate operational safety, traffic capacity, or favorable atmospheric conditions.

### 4.2. Identification results of ULATCs

Fig 9 illustrates the initial streamlines traced from boundary seed points under the four imposed directions. The streamlines generally follow relatively high-magnitude regions and form continuous paths around the modeled obstacles. White streamlines satisfy the geometric candidate criteria of length greater than 2.5 km and straightness greater than 0.75. In this case, east–west candidates are relatively concentrated near continuous open strips that visually coincide with several major arterial-road spaces, whereas north–south candidates are more dispersed. This spatial interpretation is specific to the adopted obstacle map and was not evaluated as a causal relationship with road infrastructure.

**Fig 9:**
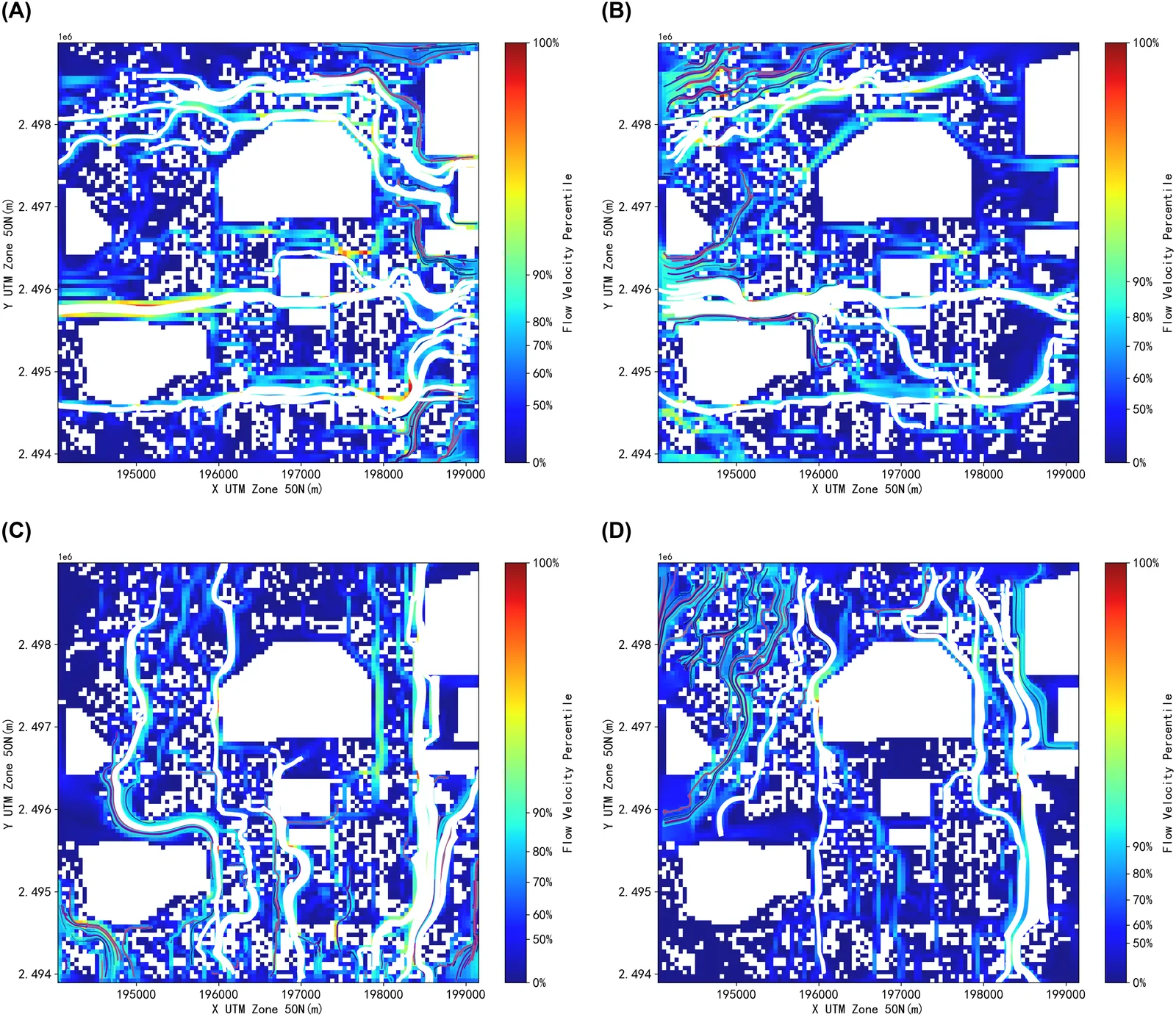
Author-generated streamline tracing results under four imposed inflow directions: (A) east, (B) west, (C) south, and (D) north. The color scale indicates relative field magnitude rather than physical wind or UAV speed.

After candidates from all four directions are pooled, redundant streamlines remain, as shown in Fig 10A. Primary clustering retains one representative from each geometrically similar group, producing the initial corridor candidates in Fig 10B. Partial overlaps between different clusters are then treated through local de-duplication, smoothing, and connection to obtain the final set in Fig 11. Qualitative overlay inspection indicates that the retained corridors are associated mainly with continuous open strips, arterial-road spaces, parks, and lower-building-density zones rather than being uniformly distributed across the study area. These associations describe the case-study morphology and do not imply that the corridors are operationally certified or accessible under all three-dimensional conditions.

**Fig 10:**
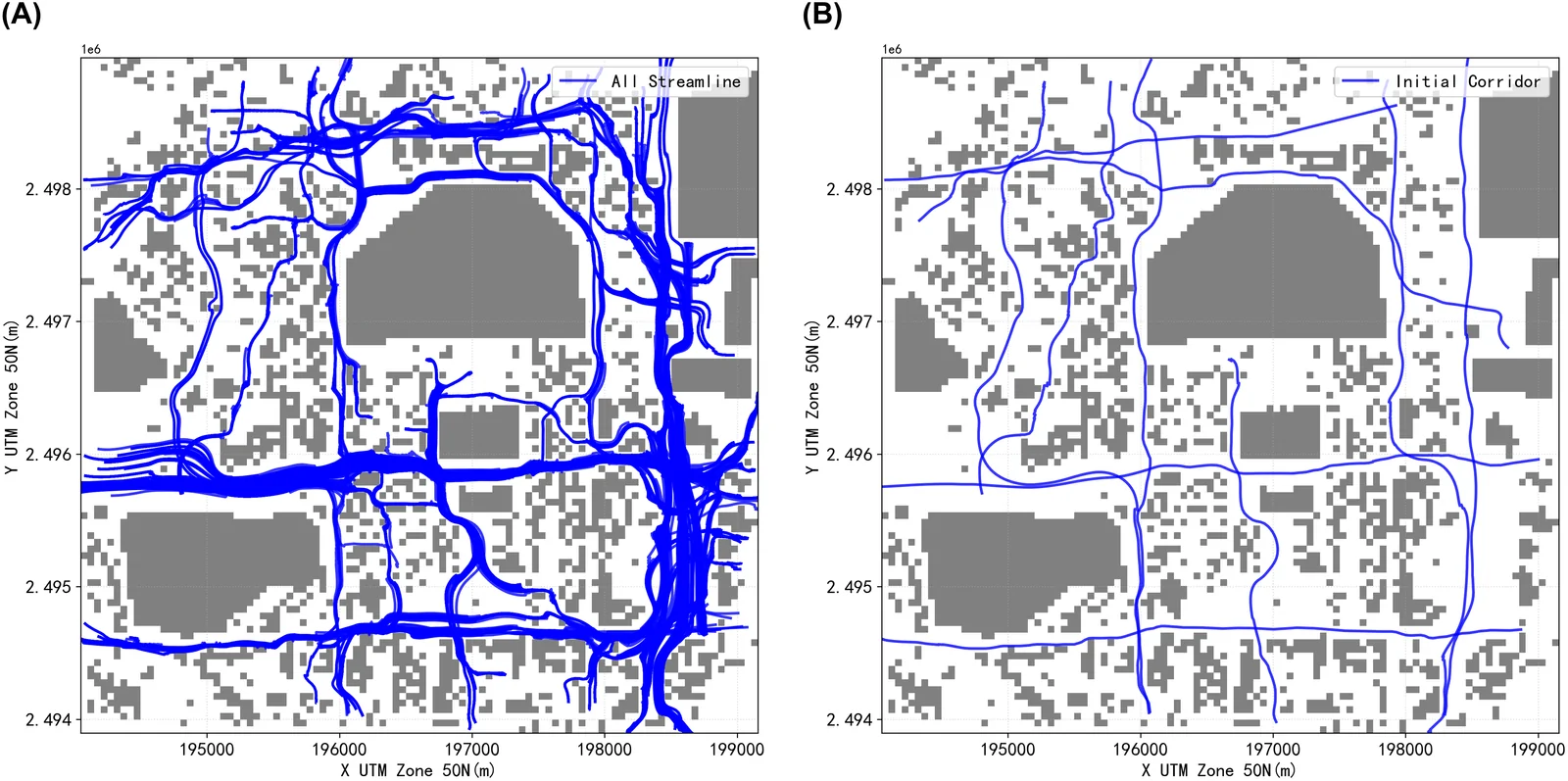
Author-generated initial streamline processing results. (A) All streamlines. (B) Initial corridors.

**Fig 11:**
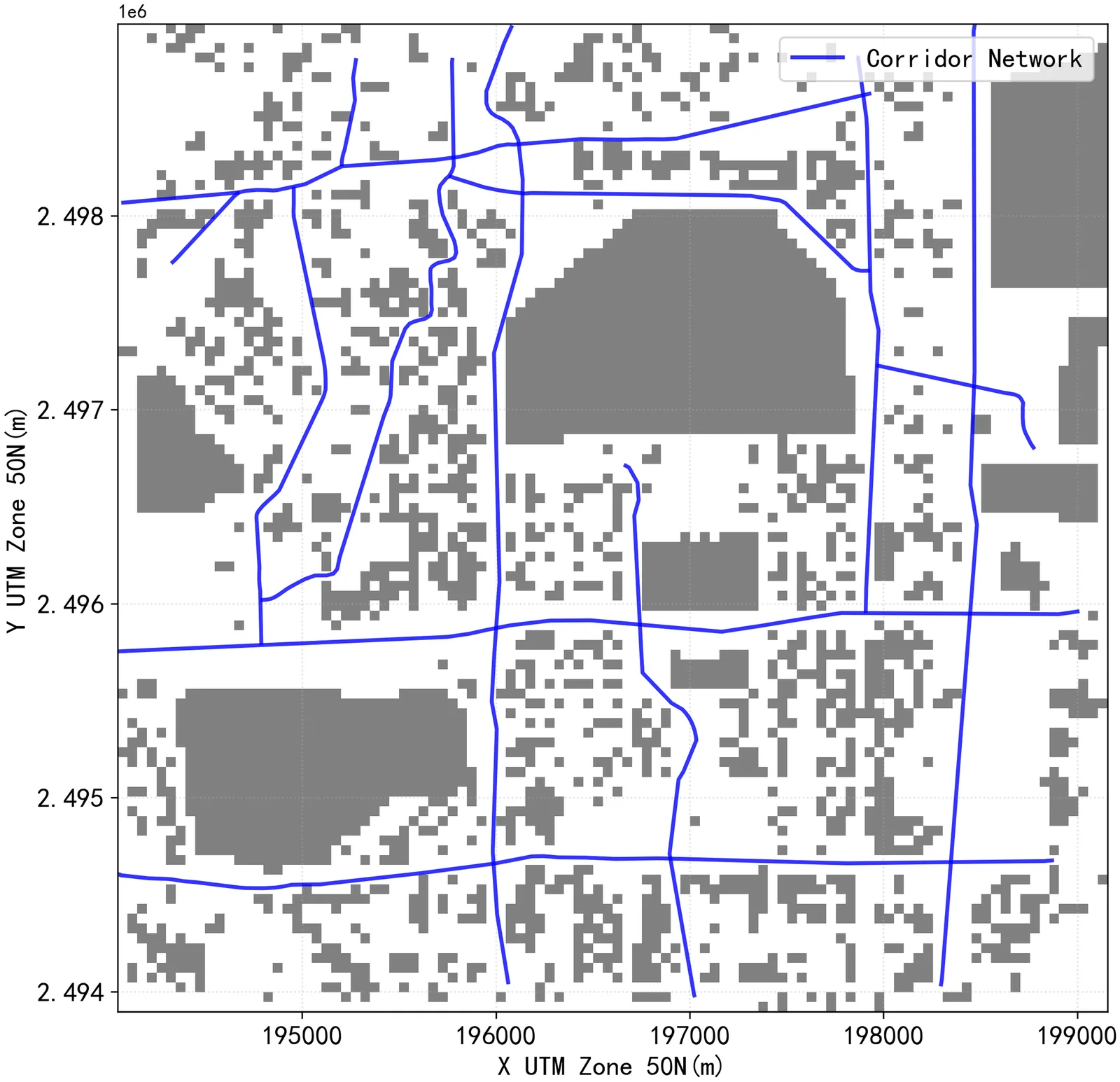
Author-generated corridor network over the processed obstacle grid.

Statistical analysis indicates that ten principal corridors were identified, with a mean individual length of 4,904 m and a mean straightness of 0.913. The sum of the individual corridor lengths was 49.043 km. After dissolving mutually overlapping segments, the unique network length was 40.271 km, corresponding to a shared-segment proportion of 17.9%.

### 4.3. Comparison with corridor-generation baselines

The proposed method was compared with two corridor-generation baselines using the same 5 km × 5 km study area, 50 m obstacle grid, and building and no-fly-zone constraints. The first baseline was adapted from the obstacle-clustered Voronoi–A* public air-route planning procedure of Zhang et al. [37]. Obstacle cells were first clustered, a Voronoi route graph was then constructed, and graph edges intersecting obstacles were repaired using grid-based A*. Spatially redundant segments were subsequently removed while preserving network connectivity, boundary access, local coverage, and obstacle clearance.

The second baseline followed the risk-trajectory aggregation concept of Slama et al. [19]. A normalized urban exposure-risk field was constructed from population, road-activity, building-exposure, and sensitive-facility data. Higher-risk cells were treated as restricted areas during baseline construction. Risk-aware trajectories were then aggregated into a reusable corridor graph, and unsafe graph edges were repaired using risk-aware A*. Small isolated components were removed after graph construction. The risk field is a relative exposure-risk proxy and should not be interpreted as an absolute accident or fatality probability.

The resulting baseline networks are shown in Fig 12, and their network characteristics are compared in Table 5.

**Table 5:** Comparison of corridor-network characteristics.

Metric	CFD-FHVCT	Voronoi–A*	Risk-GNG
Network representation	10 principal corridors	84 network segments	83 network segments
Unique network length (km)	40.271	54.484	44.555
Mean corridor/segment length (km)	4.904	0.649	0.537
Mean straightness	0.913	0.867	0.918
Length-weighted straightness	0.909	0.845	0.880
500-m coverage ratio (%)	93.55	98.48	83.90

**Fig 12:**
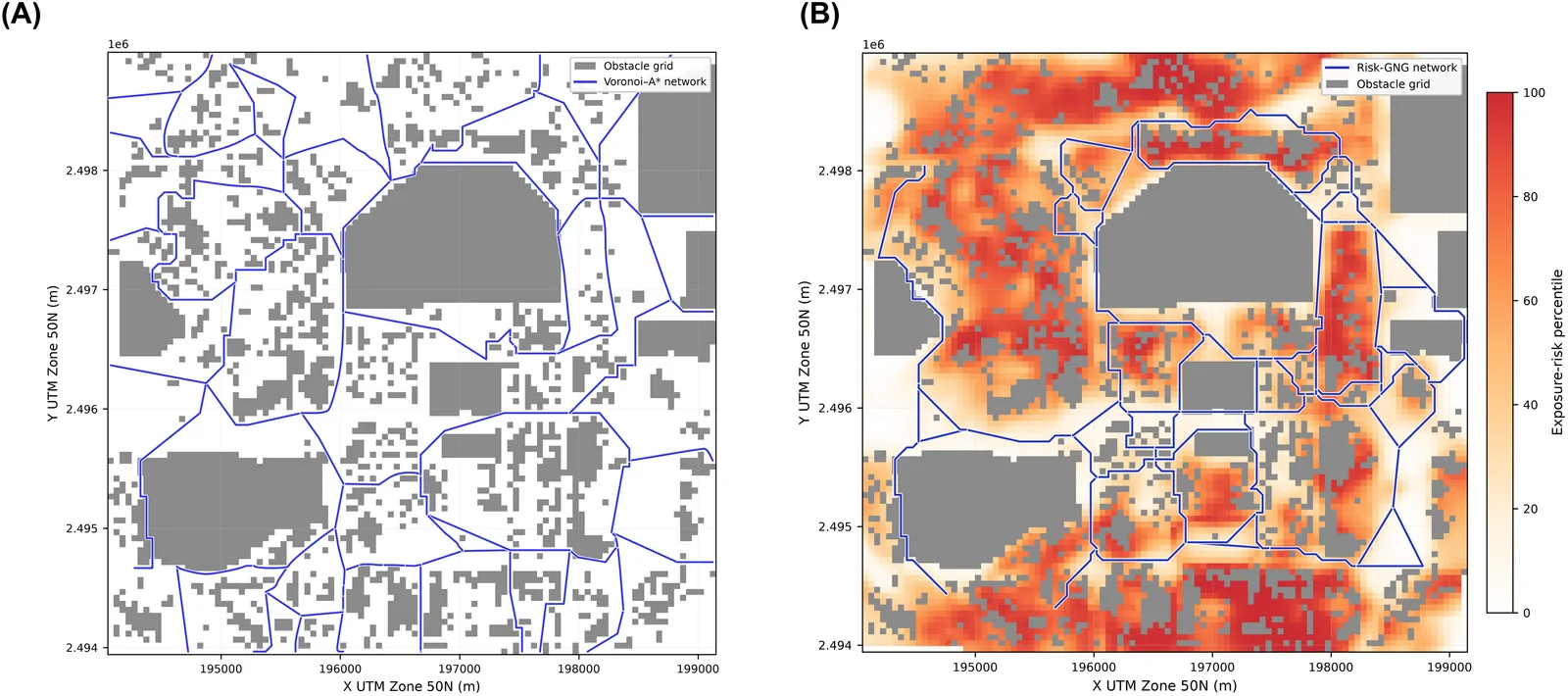
Corridor networks generated by the two baseline methods. Panel (A) shows the common obstacle grid and the Voronoi–A* network; panel (B) shows the percentile-ranked exposure-risk field and the Risk-GNG network.

Voronoi–A* achieved the highest 500-m coverage ratio (98.48%), but required the longest network (54.484 km) and had the lowest length-weighted straightness (0.845). This metric weights the straightness of each corridor or segment by its length, with values closer to 1 indicating lower overall detour. Risk-GNG produced a network length of 44.555 km and a coverage ratio of 83.90%. Its mean straightness (0.918) was slightly higher than that of CFD-FHVCT, whereas its length-weighted straightness was lower (0.880), indicating that some of its longer segments were more circuitous than suggested by the unweighted mean.

CFD-FHVCT achieved a 500-m coverage ratio of 93.55%, which was 4.93 percentage points lower than that of Voronoi–A* and 9.65 percentage points higher than that of Risk-GNG. Its unique network length was 40.271 km, which was 26.1% shorter than that of Voronoi–A* and 9.6% shorter than that of Risk-GNG. Although Risk-GNG had a marginally higher unweighted mean straightness, CFD-FHVCT achieved the highest length-weighted straightness (0.909), indicating relatively low detour along its longer principal corridors.

Overall, Voronoi–A* favored spatial coverage, whereas Risk-GNG emphasized the avoidance of high-exposure areas. CFD-FHVCT provided an intermediate coverage ratio with a shorter and less circuitous network. Because the methods use different topological representations, corridor or segment counts and their mean lengths are reported only as descriptive network characteristics.

### 4.4. Sensitivity and stability analysis

The local sensitivity analysis used the original corridor-network configuration as the baseline and examined the effects of key parameter variations on corridor count, unique network length, and straightness. The results are presented in Table 6.

**Table 6:** Sensitivity of corridor-network characteristics to parameter variations.

Configuration	Corridor count	Unique length (km)	Length-weighted straightness
Original configuration (baseline)	10	40.271	0.909
Low-speed percentile: 20	12	41.295	0.913
Low-speed percentile: 40	8	34.699	0.900
Primary buffer: 200 m	12	46.105	0.908
Primary buffer: 300 m	9	41.465	0.906
Primary similarity: 0.42	9	41.060	0.892
Primary similarity: 0.62	13	45.165	0.903
Minimum straightness: 0.70	13	46.633	0.902
Minimum straightness: 0.80	9	37.763	0.913
Grid resolution: 25 m	14	41.529	0.928
Grid resolution: 75 m	3	16.264	0.895
Speed-priority tracing weights	12	48.990	0.894
Direction-priority tracing weights	12	45.272	0.897
Synthetic inflow strength: 90%	10	41.122	0.895
Synthetic inflow strength: 110%	10	39.774	0.898

The low-speed percentile mainly affected corridor retention and network extent. The 20th-percentile setting retained 12 corridors with a unique network length of 41.295 km, whereas the 40th-percentile setting reduced these values to 8 corridors and 34.699 km. This change reflects the influence of the low-speed threshold on the spatial extent of candidate regions. Both straightness measures differed from the baseline by less than 0.01, indicating a limited effect on network geometry.

The primary-clustering parameters controlled the consolidation of nearby corridor candidates. Increasing the buffer distance from 200 to 300 m reduced the corridor count from 12 to 9 and the unique network length from 46.105 to 41.465 km. This is consistent with a larger buffer allowing more nearby candidates to be grouped. Length-weighted straightness remained nearly unchanged at 0.908 and 0.906. By contrast, increasing the similarity threshold from 0.42 to 0.62 required stronger geometric agreement for merging, resulting in more retained corridors and a longer network.

Raising the minimum-straightness threshold from 0.70 to 0.80 reduced the corridor count from 13 to 9 and the network length from 46.633 to 37.763 km, while increasing length-weighted straightness from 0.902 to 0.913. A stricter threshold therefore removed more circuitous candidates, but also reduced the overall network extent.

Grid resolution had the strongest effect. The 25-m grid retained 14 corridors with a unique network length of 41.529 km, whereas the 75-m grid retained only 3 corridors and reduced the network length to 16.264 km. In the dense urban environment considered here, coarse rasterization increases the discrete spatial extent of obstacles and compresses the feasible space between buildings. Some narrow passages may therefore become disconnected, preventing the simulated flow from passing smoothly and forming stable streamlines. This reduces both the number and total extent of the identified corridors.

The two alternative tracing-weight settings both produced 12 corridors, with unique network lengths ranging from 45.272 to 48.990 km and only moderate changes in straightness. These weights mainly adjust the relative importance of speed and direction during tracing without changing the underlying flow field or spatial constraints, which explains their comparatively limited influence on network geometry.

Overall, corridor count and network extent varied more substantially than the straightness measures across the tested configurations. Grid resolution produced the most pronounced changes, while the other parameter variations mainly affected corridor retention and network extent. Within the tested ranges, network scale and corridor composition were therefore more parameter-dependent than overall geometric directness.

The consistency of the four-direction construction was additionally examined by simultaneously setting the synthetic inflow strength to 90% and 110% of its nominal value while retaining the same obstacle grid, random seed, seed-generation rule, tracing parameters, and merging procedure. Both perturbation settings retained 10 primary corridors. Their unique network lengths were 41.122 and 39.774 km, respectively, corresponding to a relative difference of 3.3% between the two perturbed networks, while their length-weighted straightness values were 0.895 and 0.898. After buffering both networks by the 200 m primary-clustering distance, their spatial Jaccard overlap was 0.831. This paired perturbation check indicates that the dominant spatial support was retained under the tested inflow change, although local branches and network extent were not identical. It should therefore be interpreted as a limited numerical-stability check of the synthetic-field construction rather than evidence of robustness to atmospheric wind, traffic demand, or other real-world disturbances.

### 4.5. Path planning comparative analysis

Two path-planning comparison experiments were conducted. The first compared the CFD-FHVCT corridor mode with unstructured free flight to examine the operational effects of using a corridor network. The second compared CFD-FHVCT with two baseline corridor-generation methods to evaluate how different corridor networks affected the path-planning results.

#### 4.5.1. Operational comparison with unstructured free flight.

Four OD points were selected at different locations in the study area, producing six unordered OD pairs that covered east–west, north–south, and diagonal movements. A* was used to query both the proposed corridor network and the unstructured navigable grid. Route performance was evaluated using path length, mean speed, total curvature change, and flight time. The calculations represent idealized single-UAV operations without wind disturbances, traffic conflicts, or dynamic restrictions. Therefore, the results primarily reflect the tested OD cases and cannot yet support city-wide statistical inference.

Fig 13 presents the path-planning results, with path color indicating instantaneous UAV speed. Feasible paths were obtained for all six OD pairs in both the corridor mode (Fig 13A) and free-flight mode (Fig 13B). The free-flight paths were less constrained by a predefined network and were generally shorter, whereas the corridor paths followed smoother and more structured passages and required fewer pronounced heading changes in most of the tested OD cases.

**Fig 13:**
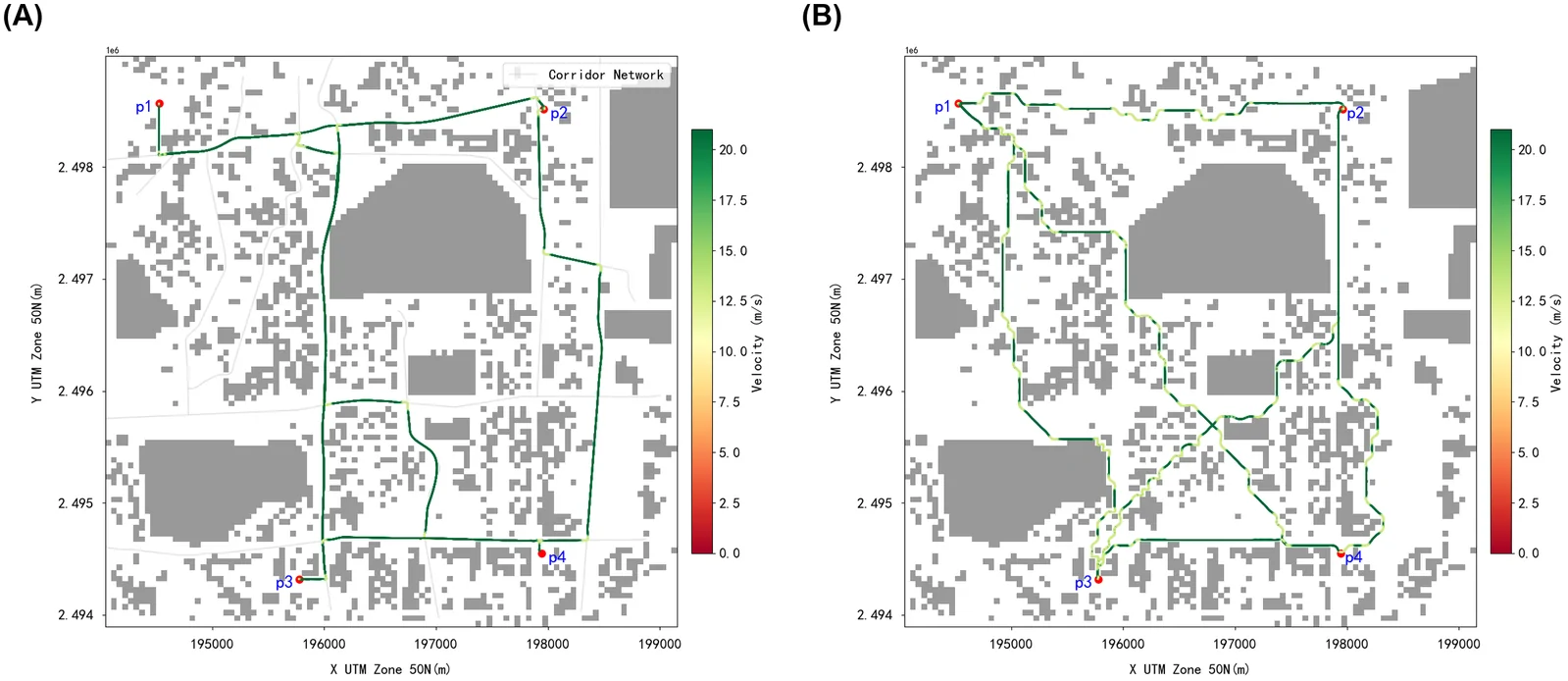
Path-planning results for (A) the proposed corridor mode and (B) the unstructured free-flight mode.

Table 7 reports the OD-level results under the baseline UAV parameter setting. The baseline values were selected with reference to the DJI Air 3S because its publicly available performance specifications provide a relatively complete basis for defining a reproducible parameter set. The baseline setting used a maximum speed of 21 m/s, a turn-limited speed of 13 m/s, and a maximum acceleration/deceleration magnitude of 7 m/s^2^. These values serve as a common reference for the comparative evaluation and are not intended to represent the full range of urban logistics UAVs.

**Table 7:** Path-planning comparison under the baseline UAV parameter setting.

OD	Path length (m)		Average speed (m/s)		Total curvature		Flight time (s)	
	Corridor mode	Free- flight	Corridor mode	Free- flight	Corridor mode	Free- flight	Corridor mode	Free- flight
1-2	4032	3636	20.5	17.7	0.83	1.16	196	205
1-3	6330	5514	20.2	16.8	1.86	4.21	313	328
1-4	7941	6228	20.3	16.8	2.14	2.38	392	370
2-3	6250	5680	20.4	16.8	0.69	3.39	306	338
2-4	4834	4561	19.8	18.3	0.85	1.00	244	249
3-4	2739	2424	20.8	18.8	1.03	0.69	132	129
**Average**	5354	4674	20.3	17.5	1.23	2.14	264	270
**Comparison**	14.6%		15.9%		−42.3%		−2.2%	

Compared with free flight, the proposed corridor mode increased mean path length by 14.6% but reduced mean total curvature change by 42.3%. Mean speed increased by 15.9%, and mean flight time decreased by 2.2%. The reduction in flight time was an average result rather than a consistent improvement across all OD pairs, as the corridor mode required slightly more time for OD pairs 1–4 and 3–4. Overall, the tested cases suggest that the corridor mode can reduce maneuvering demand and improve trajectory smoothness, although the results do not directly quantify collision risk, energy consumption, or noise.

To examine whether these findings depended strongly on the selected UAV parameters, the stored paths were re-evaluated under three kinematic scenarios (Table 8). The conservative scenario represented a lower-performance platform, the baseline scenario retained the parameter setting used in the main comparison, and the high-performance scenario represented a faster platform with greater acceleration capability. Only the kinematic parameters were changed, while the path geometry and curvature records remained fixed.

**Table 8:** Effect of UAV kinematic parameters on the corridor and free-flight paths.

Scenario	Max. speed (m/s)	Turn speed (m/s)	Acceleration (m/s^2^)	Flight time (s)		Realized speed (m/s)	
				Corridor	Free flight	Corridor	Free flight
Conservative	15	10	3	367.4	369.1	14.42	12.93
Baseline	21	13	7	263.7	270.3	20.10	17.69
High performance	25	16	8	221.1	224.0	23.97	21.33

Across the three scenarios, the differences in mean flight time between the two modes were small, with the corridor mode showing an advantage of 0.5%–2.4%. However, because the corridor paths were smoother and spent less time under turn-related speed limitations, their mean realized speed was 11.5%–13.6% higher than that of free flight. This indicates that the main benefit of the corridor mode was an increase in realized speed rather than a substantial reduction in flight time.

#### 4.5.2 Path-planning comparison among corridor-generation methods.

The second experiment compared CFD-FHVCT, Grid–Voronoi, and Risk-GNG under the same OD points, access-path procedure, graph-search method, and baseline UAV parameters. Short access paths between the OD points and each network were included in the reported route lengths. The path-planning results for the three corridor-generation methods are summarized in Table 9.

**Table 9:** Path-planning comparison among corridor-generation methods. Values are reported as the mean ± standard deviation across the six tested OD pairs. The relative-difference rows are calculated as (CFD−FHVCT−baseline)/baseline×100%. Positive values indicate that CFD-FHVCT produced a higher value, whereas negative values indicate a lower value.

Network	Path length (m)	Average speed (m/s)	Total curvature (rad)	Flight time (s)
CFD-FHVCT	5246.6±1830.1	18.60±0.63	12.49±4.95	281.8±99.7
Grid–Voronoi	5569.6±1644.9	17.02±0.62	24.58±5.39	327.5±94.6
Relative difference	−5.8%	+9.3%	−49.2%	−14.0%
Risk-GNG	6266.2±2386.9	16.21±0.41	32.45±13.78	388.2±149.5
Relative difference	−16.3%	+14.7%	−61.5%	−27.4%

For the six tested OD pairs, CFD-FHVCT produced the shortest mean route among the three structured networks, with a mean length of 5,246.6 m, compared with 5,569.6 m for Grid–Voronoi and 6,266.2 m for Risk-GNG. Relative to Grid–Voronoi, the CFD-FHVCT routes were 5.8% shorter and had 49.2% lower total curvature. Relative to Risk-GNG, the corresponding reductions were 16.3% and 61.5%.

CFD-FHVCT also had the highest mean realized speed and the shortest mean flight time in this OD sample. Its mean flight time was 14.0% lower than that of Grid–Voronoi and 27.4% lower than that of Risk-GNG. The longer and more curved Risk-GNG routes observed here are consistent with its emphasis on avoiding higher-exposure areas rather than minimizing geometric distance alone.

Because route geometry was held fixed within each network, changing the UAV kinematic parameters did not alter path length or total curvature. As an additional check on the speed- and time-based results, the three networks were evaluated under the conservative and high-performance scenarios used in Table 8. Their mean-flight-time ordering remained unchanged under both settings: CFD-FHVCT was followed by Grid–Voronoi and then Risk-GNG. Thus, the ordering observed under the baseline parameters was not reversed by the tested platform assumptions, although the magnitude of the time differences varied.

Overall, the six tested OD pairs showed descriptive differences in route directness, trajectory smoothness, and flight efficiency among the three networks. These results characterize the selected case and should not be interpreted as statistical evidence of general superiority.

## 5. Discussion

The spatial distribution of the extracted corridors indicates that the CFD-inspired formulation tends to preserve continuous open urban spaces, including arterial-road spaces, parks, and areas with relatively low building density. This correspondence is consistent with the intended role of the synthetic velocity field, which is used to identify planar continuity around obstacles rather than to represent atmospheric wind or urban UAV traffic. The comparison with Voronoi–A* and Risk-GNG also highlights different network-design priorities. Voronoi–A* may be more suitable when spatial coverage is the primary objective, whereas Risk-GNG favors the avoidance of higher-exposure areas. CFD-FHVCT, in comparison, yields a more compact network while maintaining relatively smooth trajectories. These results highlight the different priorities of the three methods in terms of spatial coverage, network compactness, and geometric directness in the tested area.

The path-planning results indicate that the main benefit of the identified network lies in reducing route curvature and maneuvering demand, while no consistent advantage in route length or flight time was observed across the six OD pairs. The reported standard deviations summarize variation among the six OD pairs rather than uncertainty from repeated experiments. The sensitivity analysis showed that grid resolution had the strongest influence on the extracted network, as coarser obstacle discretization could eliminate narrow passages. Other tested parameters mainly affected local branches and the spatial extent of the network, while the locations and overall configuration of the main corridors remained largely unchanged.

The present study evaluates a two-dimensional screening framework in a single 5×5 km^2^ urban case. The resulting evidence is therefore case-specific, and broader applicability should be examined in larger and geographically diverse urban areas. The current formulation focuses on static planar obstacle topology, while operational applications would also require high-resolution three-dimensional representation of thin or temporary obstacles, together with weather, dynamic airspace restrictions, corridor capacity, traffic demand, and multi-UAV conflict management. These factors provide natural directions for extending the framework toward three-dimensional, time-dependent, and capacity-aware operational planning. Accordingly, the identified corridors are best regarded as a strategic spatial backbone for subsequent operational assessment and should be verified against site-specific three-dimensional environmental information and current airspace constraints before practical deployment.

## 6. Conclusion

This study proposed a CFD-inspired and origin–destination-independent method for extracting candidate urban low-altitude corridor centerlines from synthetic incompressible velocity fields constrained by a two-dimensional obstacle topology. In the Futian case study, the method identified 10 principal corridors with a mean individual length of 4,904 m, a mean straightness of 0.913, and a unique network length of 40.271 km, with the resulting network mainly distributed along continuous open urban spaces.

Compared with the Voronoi–A* and Risk-GNG baseline methods, CFD-FHVCT produced the shortest unique network and the highest length-weighted straightness, with a 500-m coverage ratio of 93.55%. Across the six OD pairs, the main path-planning benefit was reduced trajectory curvature and maneuvering demand rather than consistent reductions in route length or flight time.

Overall, the results demonstrate the feasibility of CFD-FHVCT as a computational framework for strategic urban low-altitude corridor identification and candidate-network design. The identified network provides a spatial backbone for subsequent operational assessment, and future work can extend the framework toward three-dimensional and operationally constrained urban airspace applications.

## References

[1] Qu X, Zeng Z, Wang K, Wang S. Replacing urban trucks via ground–air cooperation. Communications in Transportation Research. 2022;2:100080. doi: 10.1016/j.commtr.2022.100080

[2] Wu Y, Low KH, Hu X. Trajectory-based flight scheduling for AirMetro in urban environments by conflict resolution. Transportation Research Part C: Emerging Technologies. 2021;131:103355. doi: 10.1016/j.trc.2021.103355

[3] Yan M, Yu P, Chan CA, Gygax AF, Li C, I C-L. Intelligent and Efficient UAV Delivery for Low-Altitude Economy Networking: Path Planning and Energy Consumption Optimization. IEEE Commun Mag. 2026;64(6):96–102. doi: 10.1109/mcom.001.2500729

[4] Yan M, Yu P, Chen J, Li C, I C-L. Dynamic Computation Offloading Optimization Based on Meta-Reinforcement Learning in UAV-Assisted MEC Networks. IEEE Internet Things J. 2026;13(11):23615–27. doi: 10.1109/jiot.2026.3670318

[5] Plioutsias A, Karanikas N, Chatzimihailidou MM. Hazard Analysis and Safety Requirements for Small Drone Operations: To What Extent Do Popular Drones Embed Safety?. Risk Anal. 2018;38(3):562–84. doi: 10.1111/risa.12867 28768049

[6] Wu Y, Yin H, Chen X, Zhang M. Multicircuit Route Planning for UAVs Performing the Terrain Coverage Task. IEEE Internet Things J. 2024;11(13):23765–79. doi: 10.1109/jiot.2024.3386125

[7] Cummings C, Mahmassani H. Airspace congestion, flow relations, and 4-D fundamental diagrams for advanced urban air mobility. Transportation Research Part C: Emerging Technologies. 2024;159:104467. doi: 10.1016/j.trc.2023.104467

[8] Sunil E, Ellerbroek J, Hoekstra JM, Maas J. Three-dimensional conflict count models for unstructured and layered airspace designs. Transportation Research Part C: Emerging Technologies. 2018;95:295–319. doi: 10.1016/j.trc.2018.05.031

[9] Haddad J, Mirkin B, Assor K. Traffic flow modeling and feedback control for future Low-Altitude Air city Transport: An MFD-based approach. Transportation Research Part C: Emerging Technologies. 2021;133:103380. doi: 10.1016/j.trc.2021.103380

[10] Weng C, Pan T, Chen C, Zhong R. Urban low-altitude air transport management: bridging dynamic traffic control and static network equilibrium. Transportation Research Part C: Emerging Technologies. 2025;178:105237. doi: 10.1016/j.trc.2025.105237

[11] Safadi Y, Geroliminis N, Haddad J. Integrated departure and boundary control for low-altitude air city transport systems. Transportation Research Part B: Methodological. 2024;189:103020. doi: 10.1016/j.trb.2024.103020

[12] Asslouj AE, Uppaluru H, Ramezani M, Atkins E, Rastgoftar H. A Fixed Air Corridor Model for UAS Traffic Management in Urban Areas. IEEE Trans Aerosp Electron Syst. 2024;60(5):5651–62. doi: 10.1109/taes.2024.3396788

[13] Bauranov A, Rakas J. Designing airspace for urban air mobility: A review of concepts and approaches. Progress in Aerospace Sciences. 2021;125:100726. doi: 10.1016/j.paerosci.2021.100726

[14] Cohen AP, Shaheen SA, Farrar EM. Urban Air Mobility: History, Ecosystem, Market Potential, and Challenges. IEEE Transactions on Intelligent Transportation Systems. 2021;22(9):6074–87. doi: 10.1109/TITS.2021.3082767

[15] Lau T-K, Tsai P-C, Ou H-Y, Lin T-P. Efficient and cost-effective method for identifying urban ventilation corridors using a heuristic search algorithm. Sustainable Cities and Society. 2024;101:105144. doi: 10.1016/j.scs.2023.105144

[16] Liu R, Wang Y, Zhang Y, Peng Z, Chen H, Li X, et al. Analysis of the city-scale wind environment and detection of ventilation corridors in high-density metropolitan areas based on CFD method. Urban Climate. 2025;59:102274. doi: 10.1016/j.uclim.2024.102274

[17] Yang Z. Food delivery by drone is just part of daily life in Shenzhen. MIT Technology Review. 2023.

[18] Zhai W, Han B, Li D, Duan J, Cheng C. A low-altitude public air route network for UAV management constructed by global subdivision grids. PLoS One. 2021;16(4):e0249680. doi: 10.1371/journal.pone.0249680 33852616 PMC8046233

[19] Sláma J, Váňa P, Faigl J. Generating Safe Corridors Roadmap for Urban Air Mobility. In: 2022 IEEE/RSJ International Conference on Intelligent Robots and Systems (IROS), 2022. 11866–71. 10.1109/iros47612.2022.9981326

[20] Stuive L, Gzara F. Airspace network design for urban UAV traffic management with congestion. Transportation Research Part C: Emerging Technologies. 2024;169:104882. doi: 10.1016/j.trc.2024.104882

[21] He X, He F, Li L, Zhang L, Xiao G. A route network planning method for urban air delivery. Transportation Research Part E: Logistics and Transportation Review. 2022;166:102872. doi: 10.1016/j.tre.2022.102872

[22] He X, Li L, Mo Y, Huang J, Qin SJ. A distributed route network planning method with congestion pricing for drone delivery services in cities. Transportation Research Part C: Emerging Technologies. 2024;160:104536. doi: 10.1016/j.trc.2024.104536

[23] Hughes RL. A continuum theory for the flow of pedestrians. Transportation Research Part B: Methodological. 2002;36(6):507–35. doi: 10.1016/s0191-2615(01)00015-7

[24] Wang Q. Research on Subway Passenger Flow Model Based on Fluid Mechanics Theory [Master’s thesis]. Liaoning, China: Dalian Jiaotong University; 2018.

[25] Lighthill MJ, Whitham GB. On kinematic waves I. Flood movement in long rivers. Proceedings of the Royal Society of London Series A Mathematical and Physical Sciences. 1955;229(1178):281–316. doi: 10.1098/rspa.1955.0088

[26] Li R. Research on passenger flow model at subway platform based on fluid mechanics theory. Beijing, China: Beijing Jiaotong University. 2012.

[27] Dong S, Zhang H, Li S, Jia N, He N. A Study on Urban Traffic Congestion Pressure Based on CFD. Sustainability. 2024;16(24):10911. doi: 10.3390/su162410911

[28] Rastgoftar H, Emadi H, Atkins EM. A Finite-State Fixed-Corridor Model for UAS Traffic Management. IEEE Trans Intell Transport Syst. 2024;25(3):2322–30. doi: 10.1109/tits.2023.3324250

[29] Civil Aviation Administration of China. National Airspace Basic Classification Method. Beijing, China: Civil Aviation Administration of China. 2023. https://www.caac.gov.cn/PHONE/XXGK_17/XXGK/TZTG/202312/t20231221_222397.html

[30] Che Y, Li X, Liu X, Wang Y, Liao W, Zheng X. 3D-GloBFP: The First Global Three-Dimensional Building Footprint Dataset. Earth System Science Data. 2024;16(11):5357–74. doi: 10.5194/essd-16-5357-2024

[31] Holl P, Thuerey N. Eqn148: Differentiable simulations for PyTorch, TensorFlow and Jax. In: Proceedings of the 41st International Conference on Machine Learning, 2024. 18515–46. https://proceedings.mlr.press/v235/holl24a.html

[32] Burtsev SV, Kuzmin YeP. An efficient flood-filling algorithm. Computers & Graphics. 1993;17(5):549–61. doi: 10.1016/0097-8493(93)90006-u

[33] MacCormack RW. The Effect of Viscosity in Hypervelocity Impact Cratering. Frontiers of Computational Fluid Dynamics 2002. World ScientifiC. 2001. p. 27–43. doi: 10.1142/9789812810793_0002

[34] Bates JR, McDonald A. Multiply-Upstream, Semi-Lagrangian Advective Schemes: Analysis and Application to a Multi-Level Primitive Equation Model. Mon Wea Rev. 1982;110(12):1831–42. doi: 10.1175/1520-0493(1982)110<1831:muslas>2.0.co;2

[35] Press WH, Teukolsky SA, Vetterling WT, Flannery BP. Numerical Recipes 3rd Edition: The Art of Scientific Computing. 3rd ed. Cambridge, UK: Cambridge University Press. 2007.

[36] Lee JG, Han J, Whang KY. Trajectory clustering: a partition-and-group framework. In: Proceedings of the 2007 ACM SIGMOD International Conference on Management of Data, 2007. 593–604. 10.1145/1247480.1247546

[37] Zhang H, Tian T, Feng O, Wu S, Zhong G. Research on Public Air Route Network Planning of Urban Low-Altitude Logistics Unmanned Aerial Vehicles. Sustainability. 2023;15(15):12021. doi: 10.3390/su151512021

